# The *Verticillium dahliae* Small Cysteine-Rich Protein VdSCP23 Manipulates Host Immunity

**DOI:** 10.3390/ijms24119403

**Published:** 2023-05-28

**Authors:** Jie Wang, Dan Wang, Xiaobin Ji, Jun Wang, Steven J. Klosterman, Xiaofeng Dai, Jieyin Chen, Krishna V. Subbarao, Xiaojuan Hao, Dandan Zhang

**Affiliations:** 1 College of Plant Protection, Shanxi Agricultural University, Taigu, Jinzhong 030801, China; wj876106184@163.com; 2 The State Key Laboratory for Biology of Plant Diseases and Insect Pests, Institute of Plant Protection, Chinese Academy of Agricultural Sciences, Beijing 100193, China; wangdan_star@163.com (D.W.); jixiaobin1@163.com (X.J.); wangjun32213009@163.com (J.W.); daixiaofeng_caas@126.com (X.D.); chenjieyin@caas.cn (J.C.); 3 Crop Improvement and Protection Research Unit, United States Department of Agriculture, Agricultural Research Service, Salinas, CA 93905, USA; steve.klosterman@ars.usda.gov (S.J.K.); kvsubbarao@ucdavis.edu (K.V.S.); 4 Western Agricultural Research Center, Chinese Academy of Agricultural Sciences, Changji 831100, China; 5 Department of Plant Pathology, University of California, Davis, c/o U.S. Agricultural Research Station, Salinas, CA 93905, USA

**Keywords:** *Verticillium dahlia*, small cysteine-rich proteins, inhibiting plant immunity, subcellular localization, virulence

## Abstract

Verticillium wilt caused by *Verticillium dahliae* is a notorious soil-borne fungal disease and seriously threatens the yield of economic crops worldwide. During host infection, *V. dahliae* secretes many effectors that manipulate host immunity, among which small cysteine-rich proteins (SCPs) play an important role. However, the exact roles of many SCPs from *V. dahliae* are unknown and varied. In this study, we show that the small cysteine-rich protein VdSCP23 inhibits cell necrosis in *Nicotiana benthamiana* leaves, as well as the reactive oxygen species (ROS) burst, electrolyte leakage and the expression of defense-related genes. VdSCP23 is mainly localized in the plant cell plasma membrane and nucleus, but its inhibition of immune responses was independent of its nuclear localization. Site-directed mutagenesis and peptide truncation showed that the inhibition function of VdSCP23 was independent of cysteine residues but was dependent on the *N*-glycosylation sites and the integrity of VdSCP23 protein structure. Deletion of *VdSCP23* did not affect the growth and development of mycelia or conidial production in *V. dahliae*. Unexpectedly, *VdSCP23* deletion strains still maintained their virulence for *N. benthamiana*, *Gossypium hirsutum* and *Arabidopsis thaliana* seedlings. This study demonstrates an important role for VdSCP23 in the inhibition of plant immune responses; however, it is not required for normal growth or virulence in *V. dahliae*.

## 1. Introduction

Phytopathogenic fungi have evolved complex mechanisms for invading hosts to obtain nutrients and reproduce, and the diseases they cause result in major economic losses worldwide. Unlike the animal kingdom, the members of which have evolved adaptive immune systems, plants have evolved innate immune systems to recognize and inhibit infection by pathogenic fungi [1]. When plant receptors recognize pathogens, they can activate a series of antimicrobial responses, including ion fluxes, accumulation of reactive oxygen species (ROS), expression of genes associated with hypersensitive response (HR) and quick activation of defense-related mitogen-activated protein kinase (MAPKs) cascades [2,3,4].

During host infection, the relatively conserved pathogen- or microbe-associated molecular patterns (PAMPs/MAMPs) can be recognized by the pattern recognition receptors (PRRs) on the plasma membrane to trigger the broad-spectrum resistance response, commonly termed PAMP (or MAMP)-triggered host immunity (PTI). To obtain nutrients and reproduce as the disease progresses, pathogens have evolved another mechanism to inhibit host immune responses. Pathogens can secrete a large number of effector proteins into plant apoplast or cytoplasm to manipulate plant immunity and, eventually, achieve successful infection [5]. Plants, therefore, have evolved corresponding resistance (R) proteins to recognize the effectors secreted by pathogens specifically and then stimulate deeper effector-triggered immunity (ETI) [6,7,8,9]. Plant PTI and ETI have synergistic effects co-regulating pathogen infection [10].

The first fungal effector was cloned in *Cladosporium fulvum* [11] and, since then, studies on different plant pathogenic fungi have shown their ubiquity in the different plant pathological systems. Effectors are a group of proteins that have little or no similarity with each other and function independently [12,13,14]. Effector proteins have been classified based on their functional characteristics, and among them, small cysteine-rich secretory proteins have been widely studied [15]. The identification of such secreted proteins has relied on the number of amino acids (≤400 aa), the number of cysteine residues (≥4 cysteine residues) and the lack of a transmembrane domain [16]. Intramolecular disulfide bonds formed by cysteine residues in small cysteine-rich proteins are crucial to the stability of protein structure [17]. The disulfide bonds also prevent the protein from being degraded by the plant protease [18]. In addition, the *N*-glycosylation of SCPs can also affect their normal function [19,20]. An increasing number of SCPs (about 100 or more) have been identified in fungi such as *Verticillium dahliae*, *Fusarium graminearum*, *Ustilago maydis*, *Ustilaginoidea virens* and *Magnaporthe oryzae* [20,21,22,23]. These SCPs manipulate immunity in different ways: Cce1 from *U. maydis* can inhibit plant immune responses by localizing in the apoplast of plants and is an important virulence factor in *U. maydis* [24], while PpEC23 from *Phakopsora pachyrhizi* can bind to GMSPL12l, a negative regulator of soybean immunity, and inhibit plant immune responses [25]. Thus, SCPs are effector proteins that play key roles in the plant–pathogen interactions.

*Verticillium dahliae* is a soil-borne pathogenic fungus that causes Verticillium wilt, mainly in dicotyledonous plants, causing significant economic losses for crops [26,27]. As early as the middle of the 20th century, researchers began to study the secreted proteins produced by *V. dahliae*, and in the later studies, some functional protein molecules were identified successively [28]. These secreted proteins target different sites and perform different functions during the infection with *V. dahliae*. These functions can be divided into the following categories: manipulation of host immunity, hormone homeostasis interference, cytotoxicity, oxidative stress neutralization, fungal nutrition, morphological development, microbiome manipulation and cell wall degradation [29]. For manipulation of host immunity, *V. dahliae* produces secreted proteins that induce host immunity and that also inhibit host immunity [30,31,32,33,34,35,36]. Similarly, the SCPs of *V. dahliae* can also induce or inhibit host immune response via different ways and methods. VdSCP7, which localizes in the nucleus of *N. benthamiana* cells, could activate salicylic acid and jasmonic acid signaling pathways to enhance resistance in *N. benthamiana* leaves against *Botrytis cinerea* and *Phytophthora capsici* [37]. VdSCP27/113/126, with receptors BAK1 and SOBIR1, can trigger immune responses in *N. benthamiana* [23]. VdSCP76 and VdSCP77, from the CFEM family, suppress immune responses triggered by certain effector proteins [38]. VdSCP41 is localized in the plant nucleus, binds to CBP60g and inhibits transcription factor activity and, in turn, the plant immune responses [39]. Therefore, the function analysis of SCPs of *V. dahliae* is helpful for us to further understand the process of interaction between *V. dahliae* and host plants. Our previous bioinformatics analysis showed that *V. dahliae* Vd991 encodes 123 SCPs, but only a few protein functions have been characterized [16,23,38,40].

In this study, we mainly focused on the functional analysis of the small cysteine-rich protein VdSCP23 in *V. dahliae*–plant interactions. The main objectives were to: (1) elucidate the secretory characteristics and subcellular localization of VdSCP23 in plant cells; (2) investigate the functional characteristics of VdSCP23 in manipulating host immunity; and (3) identify the function of VdSCP23 in *V. dahliae* growth, morphology and virulence.

## 2. Results

### 2.1. VdSCP23 Displays Broad-Spectrum Cell Death Suppression

**Figure 1:**
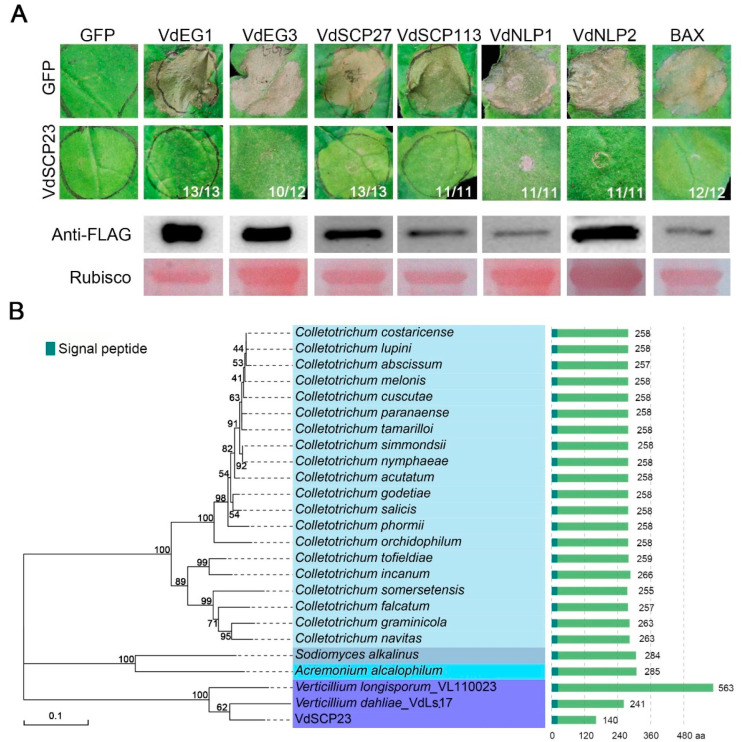
Analyses of cell death-suppression activity of *Verticillium dahliae* VdSCP23 and phylogeny of its homologs in fungi. (**A**) Suppression activity of VdSCP23 against the known cell death-inducing proteins VdEG1, VdEG3, VdSCP27, VdSCP113, VdNLP1 and VdNLP2 transiently expressed in 4-week-old *N. benthamiana* leaves. The green fluorescent protein (GFP) and BAX expression were used as controls. The tested proteins were fused with the FLAG tag. Immunoblotting analysis was performed for the transiently expressed VdEG1, VdEG3, VdSCP27, VdSCP113, VdNLP1, VdNLP2 and BAX proteins fused to the FLAG-tag in *N. benthamiana* leaves 72 h after infiltration. Ponceau *S*-stained Rubisco protein is shown as the total protein loading control. The numerator at the bottom of the leaves represents the number suppressing cell death out of the total number of tested leaves (denominator). (**B**) Phylogenetic analysis of VdSCP23 and the homologous sequences from other fungi. “aa” represents amino acid. MEGA11 software was used to construct the phylogenetic tree of homologous proteins in *Sodiomyces alkalinus*, *Acremonium alcalophilum*, *Colletotrichum* spp. and *Verticillium* spp. with the neighbor-joining method.

Previous studies have shown that VdSCP23 can inhibit cell death in tobacco leaves caused by VdEG1 [23]. To further investigate whether VdSCP23 has broad-spectrum activity in inhibiting cell death, it was co-expressed with six known cell death-inducing proteins (VdEG1, VdEG3, VdSCP27, VdSCP113, VdNLP1, VdNLP2) [23,34,35,41] and the programed cell death factor Bcl-2-associated X protein (BAX) [42], respectively. The results indicated that VdSCP23 could inhibit the cell death induced by the above PAMPs and effectors in *N. benthamiana* leaves (Figure 1A). Simultaneously, these proteins were verified as expressed by immunoblotting analysis (Figure 1A).

### 2.2. VdSCP23 Is Conserved among Strains of Verticillium dahliae

A phylogenetic analysis was performed using 206 VdSCP23 homologous protein sequences from fungi after screening out proteins (contained signal peptide; sequence similarity < 30%; sequence length < 600 aa) (Table S1). We found that the homologous proteins of VdSCP23 mainly existed in *Colletotrichum* spp., with the exception of *Verticillium longisporum*, *Sodiomyces alkalinus* and *Acremonium alcalophilum* (Figure 1B). VdSCP23 showed the most recent relationship with the predicted homologous proteins in *Verticillium* spp., *V. dahliae* Ls.17 and *V. longisporum*, and these were clustered in one branch (Figure 1B and Figure S1). However, the sequence length of VdSCP23 was significantly shortened at its C-terminus compared to the homologs in strain *V. dahliae* Ls.17 and *Verticillium longisporum* (Figure 1B and Figure S1). The sequence variation for the VdSCP23 homologs in 159 *V. dahliae* strains, all of which were derived from whole-genome sequencing of these strains in our laboratory (Verticilli-Omics: https://db.cngb.org/Verticilli-Omics/ (accessed on 5 November 2021) ), were also analyzed. There were 9 variants among the VdSCP23 homologs from the 159 *V. dahliae* strains. In six strains, the predicted protein sequence was identical to VdSCP23, belonging to variant 1 (Figure S2). The sequences in variants one, two and three were highly conserved, with only one or two amino acid substitutions. Compared to variants four, five and six, the sequence of variant one showed a premature termination to 110 amino acids in length (Figure S2 and Table S2). However, compared to variants seven, eight and nine, variant one revealed start-codon changes (Figure S2). Together, these results show that, even if VdSCP23 is relatively conserved in the genus *Verticillium*, analyses of the amino acid sequences revealed distinct polymorphisms within a population of *V. dahliae* strains, indicating potential functional differentiation.

### 2.3. VdSCP23 Is Secreted to the Extracellular Region of Verticillium dahliae

**Figure 2:**
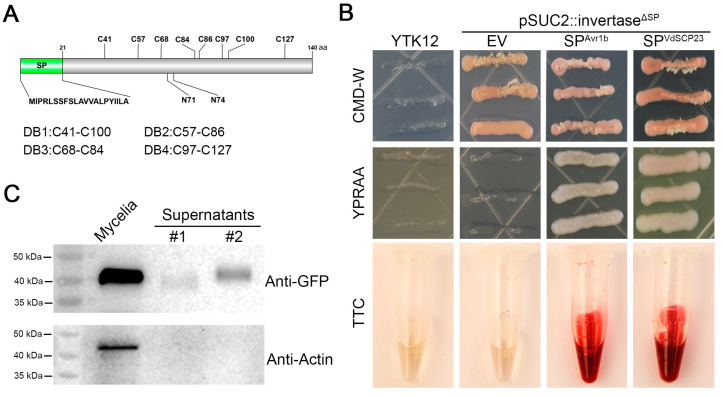
VdSCP23 possesses a signal peptide and is secreted extracellularly from *Verticillium dahliae*. (**A**) Features of the primary structures and potential crucial sites of VdSCP23. Amino acids 1–21 encode signal peptides; “C” represents a cysteine residue; “DB” represents the disulfide bond formed by cysteine; N71 and N74 represent two *N*-glycosylation modification sites. (**B**) Functional validation of the putative N-terminal signal peptide of VdSCP23 using yeast invertase secretion and 2,3-triphenyltetrazolium chloride (TTC) assays. YTK12 is an invertase secretion-deficient yeast strain. The secretion of invertase was indicated by the growth of YTK12 on YPRAA plates with raffinose as sole carbon source. TTC was used to test the enzymatic activity via its reduction to red formazan. The signal peptide of Avr1b and the empty vector were used as positive and negative controls, respectively. (**C**) VdSCP23-GFP was detected in supernatants and the total VdSCP23-GFP mycelia cell lysate via Western blotting analysis. The non-secreted actin protein was used as control and was only detected in the total thallus cell lysate. Supernatants #1 and #2 represent the supernatants of two strains of VdSCP23-GFP, respectively.

To further explore the function of VdSCP23, the basic characteristics of VdSCP23 were analyzed. VdSCP23 encodes 140 amino acids, includes 8 cysteine residues predicted to form 4 disulfide bonds and does not contain any known conserved domains (Figure 2A). VdSCP23 is predicted to be a glycoprotein with two putative *N*-glycosylation sites that are located at Asn-71 (NDTL) and Asn-74 (NDTL). As a typical SCP, VdSCP23 was also predicted to contain a signal peptide (Figure 2A). To confirm the secretory characteristics of VdSCP23, signal peptide-mediated protein secretion activity was verified using a yeast signal trap system [43] with a 2,3,5-triphenyltetrazolium chloride (TTC) catalysis assay [44]. The signal peptide of VdSCP23 had the ability to mediate secretion of invertase after its coding sequence was fused with the invertase gene lacking a signal peptide in the vector pSUC2 in yeast, which conferred on the yeast strain YTK12 the ability to utilize raffinose as a sole carbon source (Figure 2B). In addition, the yeast strain YTK12 containing the recombinant plasmid (including the signal peptide of VdSCP23 and the invertase gene lacking a signal peptide) could catalyze TTC so as to generate the red product triphenylformazan (Figure 2B). These results indicate that the signal peptide of VdSCP23 is functional and that VdSCP23 is likely secreted by *V. dahliae*. Furthermore, Western blotting showed the presence of the VdSCP23-GFP protein in the culture supernatant and in the mycelia of the VdSCP23-GFP strain. Actin was detected only in the mycelia of the VdSCP23-GFP strain but not in the culture supernatant, suggesting that VdSCP23-GFP protein was not released by cell rupture during mycelia-induced culture (Figure 2C). Taken together, the above results confirmed that VdSCP23, a small cysteine-rich protein, could be secreted to the extracellular space of *V. dahliae*.

### 2.4. VdSCP23 Is Mainly Localized on Plasma Membrane or Cytoplasm for the Exertion of Its Cell Death-Suppression Function

**Figure 3:**
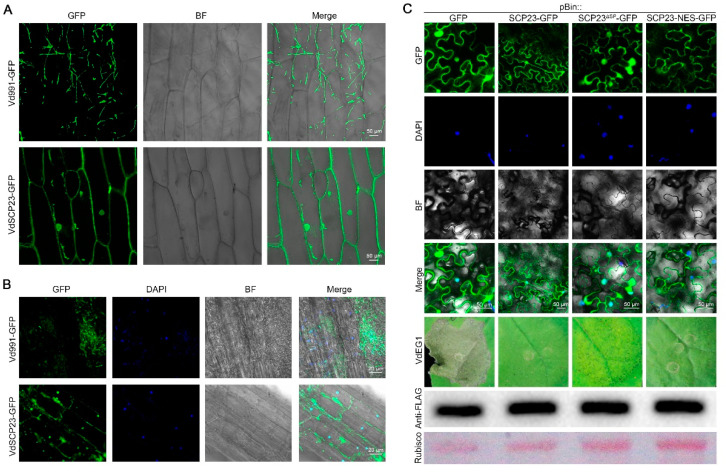
VdSCP23 from *Verticillium dahliae* localizes on the plant plasma membrane to exert its cell death suppression function. (**A**) Subcellular localization of VdSCP23 in onion epidermal cells. The conidia from the EC::*VdSCP23-GFP* strain of *V. dahliae* was co-incubated with onion epidermal cells for 5 days, while wild-type Vd991-GFP was used as a control. Bars = 50 μm. (**B**) Localization of VdSCP23 in *Arabidopsis thaliana* root cells. A conidial suspension of the EC::*VdSCP23-GFP* strain was co-incubated with *A. thaliana* root cells for 5 days, and wild-type Vd991-GFP was used as control. The fluorochrome 4’,6-diamidino-2-phenylindole (DAPI) was used as fluorescence marker for nuclei. Bars = 20 μm. (**C**) Subcellular localization of the native VdSCP23 and corresponding mutants (VdSCP23^ΔSP^ and VdSCP23-NES) fused at the *C*-terminus to GFP. The proteins were transiently expressed in 4-week-old *N. benthamiana* leaves and observed 2 days post-agroinfiltration. The vector pBin::GFP was used as a control. The fluorochrome DAPI was used as a fluorescence marker for nuclei. Bars = 50 μm. The inhibition function of VdSCP23, VdSCP23^ΔSP^ and VdSCP23-NES against VdEG1-induced cell death was assayed in 4-week-old *N. benthamiana* leaves. The efficiency of the transient expression of the cell death-inducing gene VdEG1 was validated by Western blotting with the FLAG-tag antibody. The Ponceau S-stained Rubisco protein is shown as a total protein loading control. The fluorescence was imaged with a Leica TCS SP8 confocal microscopy system with an excitation wavelength of 488 nm and an emission wavelength of 510 nm for GFP and an excitation wavelength of 340 nm and emission wavelength of 488 nm for the DAPI dye.

To further investigate the immune suppression function of VdSCP23, its subcellular location was examined in onion epidermal cells and *A. thaliana* root cells by fusing it with green fluorescent protein (GFP). Specifically, the *VdSCP23* deletion strain was obtained by replacing the targeted gene *VdSCP23* with a hygromycin resistance cassette through homologous recombination (Δ*VdSCP23*), and the *VdSCP23-GFP* was introduced into the Δ*VdSCP23* strain to obtain the complemented strain EC*VdSCP23-GFP*. Onion epidermal cells were also inoculated with the conidia of the EC*VdSCP23-GFP* strain, and the distribution of the green fluorescence signal in the epidermal cells was observed with confocal microscopy two days after inoculation. Similarly, the *A. thaliana* seedling roots were inoculated with the conidia of the EC*VdSCP23-GFP* strain, and the distribution of the green fluorescence signal in root-tip cells was observed with confocal microscopy five days after inoculation. Whether in *A. thaliana* or onion epidermal cells, the VdSCP23-GFP fusion protein was mainly concentrated along the cell plasma membrane and intercellular space, including the nuclei and cytoplasm (Figure 3A,B). The fusion protein present in nuclei was confirmed by overlapping with the fluorescence of nuclear dye 4′,6-diamidino-2-phenylindole (DAPI) (Figure 3B). All the above results indicated that VdSCP23 could be secreted into plant cells and mainly localizes in plasma membrane, nuclei and cytoplasm during *V. dahliae* infection of host plants.

To further clarify the relationship between the localization of VdSCP23 in *N. benthamiana* leaves and the roles of VdSCP23 in suppressing immunity, the native VdSCP23, VdSCP23^Δsp^ (VdSCP23 without signal peptide) and VdSCP23-NES (VdSCP23 with nuclear export signal) sequences were fused with GFP and transiently expressed in *N. benthamiana* leaves. As for the findings from *A. thaliana* and onion, the native VdSCP23 mainly localized at the *N. benthamiana* cell plasma membrane and nuclei and in the cytoplasm (Figure 3C). We further confirmed the location of VdSCP23 in the cell membrane through the plasmolysis of tobacco cells (Figure S3). VdSCP23 without a signal peptide showed similar localization as native VdSCP23, and the suppression function was also unaffected (Figure 3C). Further, we also examined the inhibitory effect of VdSCP23^Δsp^ on other cell death-inducing proteins, which still showed broad-spectrum inhibitory activity (Figure S4). When the NES sequence was fused to the N-terminus of VdSCP23, its green fluorescence signal mainly disappeared from the nuclei but accumulated near the plasma membrane and cytoplasm. Meanwhile, its cell death-inhibitory function remained normal (Figure 3C). Together, these results indicated that the function of VdSCP23 in inhibiting immunity is independent of its localization in the host nuclei and mainly dependent on its localization in plant plasma membrane or cytoplasm.

### 2.5. VdSCP23 Significantly Enhanced Host Susceptibility by Suppressing Immunity

**Figure 4:**
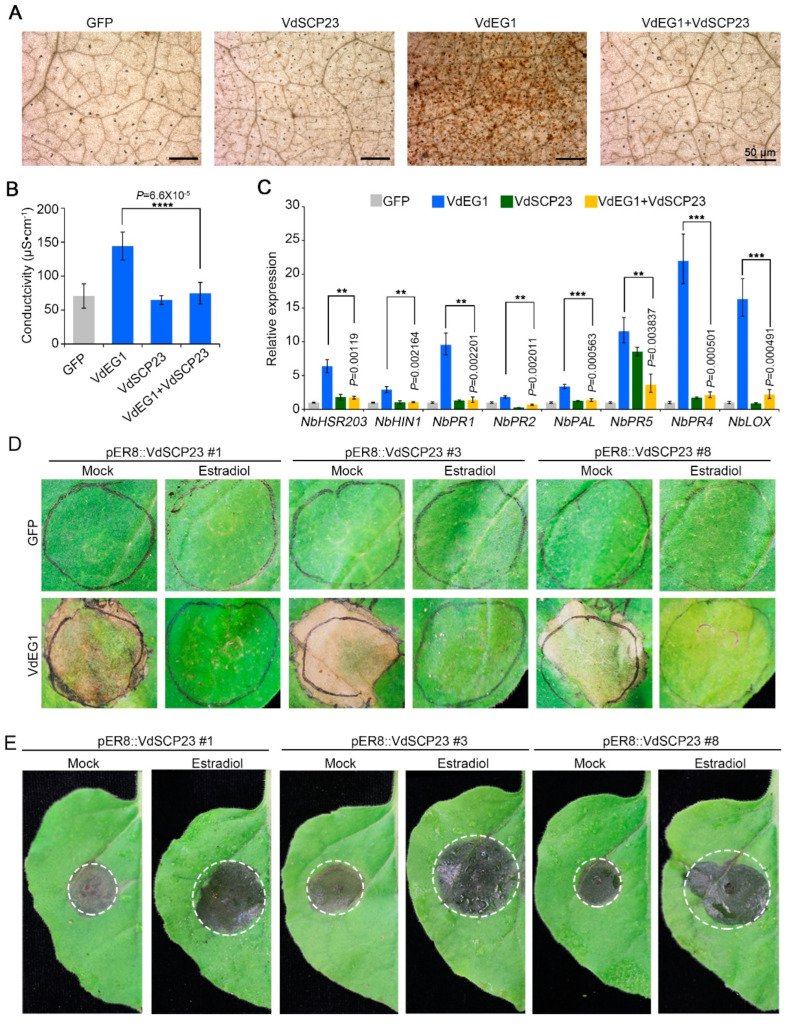
VdSCP23 from *Verticillium dahliae* enhances host susceptibility by suppressing immunity. (**A**) Reactive oxygen species (ROS) accumulation after transient co-expression of VdSCP23 with VdEG1 in 4-week-old *N. benthamiana* leaves was determined by 3,3′-diaminobenzidine (DAB) staining. (**B**) Electrolyte leakage assay in 4-week-old *N. benthamiana* leaves 48 h after co-infiltration of constructs expressing VdEG1 simultaneously with VdSCP23. Single infiltrations of VdEG1 and GFP were used as the controls. (**C**) Expression of resistance-related genes was quantified using reverse transcription quantitative PCR (RT-qPCR) 2 days after co-agroinfiltration of VdSCP23 with VdEG1 in *N. benthamiana* leaves. Single infiltrations of VdEG1 and GFP were used as controls. (**D**) Transient expression of VdEG1 in *VdSCP23* transgenic lines induced by estradiol was detected, and the non-estradiol-induced transgenic lines served as controls. The phenotypes were photographed 5 days after agro-infiltration. (**E**) Disease symptoms of *Botrytis cinerea* on *N. benthamiana* plants expressing VdSCP23. The treatments and controls were conducted with estradiol-induced and non-estradiol-induced transgenic lines, respectively. The dashed circle indicates the boundary of pathogen infestation on tobacco leaves. The error bars represent standard errors. **, *** and **** represent significant differences at *p* < 0.01, *p* < 0.001 and *p* < 0.0001 between VdEG1 and VEG1 + VdSCP23 according to Student’s *t* tests.

VdSCP23 displayed the activity to suppress cell death induced by typical PAMPs and other effectors (Figure 1A). The host immunity response triggered by PAMPs and effectors includes a reactive oxygen species (ROS) burst, electrolyte leakage and the induction of defense-related genes, which can suppress pathogen infection [3,45]. To further confirm the immune inhibitory function of VdSCP23, it was co-expressed transiently with the typical PAMP VdEG1 in *N. benthamiana* leaves. We found the ROS accumulation and the electrolyte leakage caused by VdEG1 were significantly alleviated following co-expression with VdSCP23 (Figure 4A,B). The gray value analysis of reactive oxygen species suggested the same result (Figure S5A). Correspondingly, the defense-related genes (*NbHSR203, NbHIN1, NbPR1, NbPR2, NbPAL, NbPR5, NbPR4, NbLOX*) induced by VdEG1 were significantly suppressed when co-expressed with VdSCP23 in *N. benthamiana* leaves (Figure 4C). These results indicated that VdSCP23 could effectively inhibit plant immunity induced by PAMPs.

To further explore whether the plant immunity-inhibition function of VdSCP23 can make plants more susceptible and enhance pathogen infection, we examined the susceptibility of VdSCP23 transgenic *N. benthamiana* leaves to *Botrytis cinerea.* Transgenic lines of *N. benthamiana* with VdSCP23 (pER8::VdSCP23) were generated with the estrogen-inducible XVE system (LexA-VP16-Estragon Receptor), which induces gene (*VdSCP23*) expression and a rapid accumulation of protein with the estrogen treatment [46]. pER8::VdSCP23 transgenic lines sprayed with water were used as controls. The expression of VdSCP23 in pER8::VdSCP23 transgenic lines (numbers one, three and eight) was obviously induced after treatment with estrogen (Figure S5B). pER8::VdSCP23 transgenic *N. benthamiana* induced by estradiol could still inhibit cell necrosis caused by VdEG1 in *N. benthamiana* leaves (Figure 4D). Compared to the non-estradiol-induced transgenic lines (water control), the lesion size from *B. cinerea* infection on estradiol-induced transgenic *N. benthamiana* leaves rapidly increased (Figure 4E). The lesion diameters and *B. cinerea* biomass were similarly affected (Figure S5C,D). In addition, examination of the susceptibility of *N. benthamiana* in which VdSCP23 was transiently expressed revealed that the lesion phenotypes, lesion diameters and the *B. cinerea* biomass were significantly increased in *N. benthamiana* leaves transiently expressing VdSCP23 (Figure S5E–G). Above all, we confirmed that VdSCP23 can increase plant susceptibility and facilitate pathogen infection by suppressing plant immunity.

### 2.6. N-glycosylation Sites and Protein Structural Integrity, but Not Cysteine Residues, Are Necessary for the Immunity-Inhibiting Function of VdSCP23

**Figure 5:**
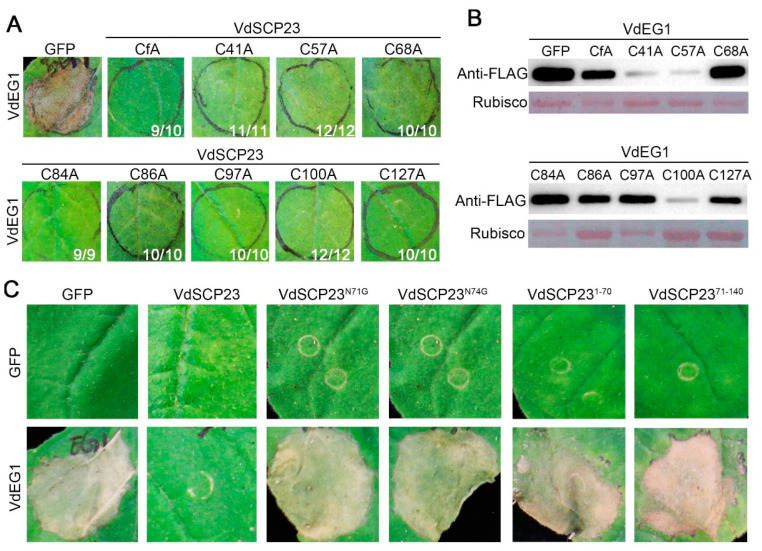
The inhibition function of VdSCP23 from *Verticillium dahliae* depends on the *N*-glycosylation sites and protein structure integrity. (**A**) The cell death-suppression activities of the wild-type and cysteine residue site-directed mutant proteins of VdSCP23 were determined via transient co-expression with VdEG1, respectively, in 4-week-old *N. benthamiana* leaves. “CfA” indicates that all cysteine residues were replaced with alanine. “C-No.-A” represents the single cysteine residue replaced with alanine in the respective position in VdSCP23. Co-expression of GFP and VdEG1 was used as a control. (**B**) The efficiency of the transient expression of VdEG1 was validated with Western blotting with a FLAG-tag. The Ponceau *S*-stained Rubisco protein is shown as a total protein loading control. (**C**) *N*-glycosylation site-directed mutagenesis and truncated proteins of VdSCP23 were co-expressed with VdEG1 in *N. benthamiana* leaves, respectively. The phenotypes were photographed 5 days after agro-infiltration. “N-No.-G” indicates the *N*-glycosylation site was replaced by glycine. Co-expression of VdEG1 with GFP or VdSCP23 served as negative and positive controls, respectively.

VdSCP23 contains eight cysteine residues that were predicted to form four potential disulfide bonds [47] (Figure 2A). To detect the activity of potential disulfide bonds in suppressing immunity, the immune-suppressive function of VdSCP23 following site-direct mutagenesis of cysteine codons in *VdSCP23* (alanine substitute for cysteine, C>>A) was assessed. The results revealed that disruption of the disulfide bond had no effect on the function of VdSCP23 whether the eight cysteine residues were substituted simultaneously or substituted separately (Figure 5A). Moreover, VdEG1 was expressed normally in each treatment, as determined by immunoblotting analysis (Figure 5B).

Glycosylation of protein after translation is a common protein modification. *N*-glycosylation of secreted proteins plays an important role in immune suppression [19,48]. VdSCP23 has two potential *N*-glycosylation sites (VdSCP23^N71^ and VdSCP23^N74^) based on the prediction of the NetNGlyc-1.0 Web server (Figure 2A) [49]. The contributions of two *N*-glycosylation sites to the immune-suppressive function of VdSCP23 were investigated. The results indicated that transient expression of VdSCP23^N71^ or VdSCP23^N74^ resulting in the cell death-suppressing activity induced by VdEG1 in *N. benthamiana* leaves being lost (Figure 5C).

To further identify the functional peptide of VdSCP23, the immunity suppression activities of two segments of VdSCP23—VdSCP23^1−70aa^ and VdSCP23^71−140aa^—were examined via co-expression with VdEG1, respectively. Whether VdSCP23^1−70aa^ or VdSCP23^71−140aa^ was co-expressed with VdEG1, the inhibitory function of VdSCP23 was lost (Figure 5C). Taken together, we confirmed that two potential *N*-glycosylation sites and the integrity of the protein structure are critical for the inhibitory function of VdSCP23.

### 2.7. VdSCP23 Was Not Required for Growth, Normal Morphology, Stress Tolerance, or Carbon Source Utilization

**Figure 6:**
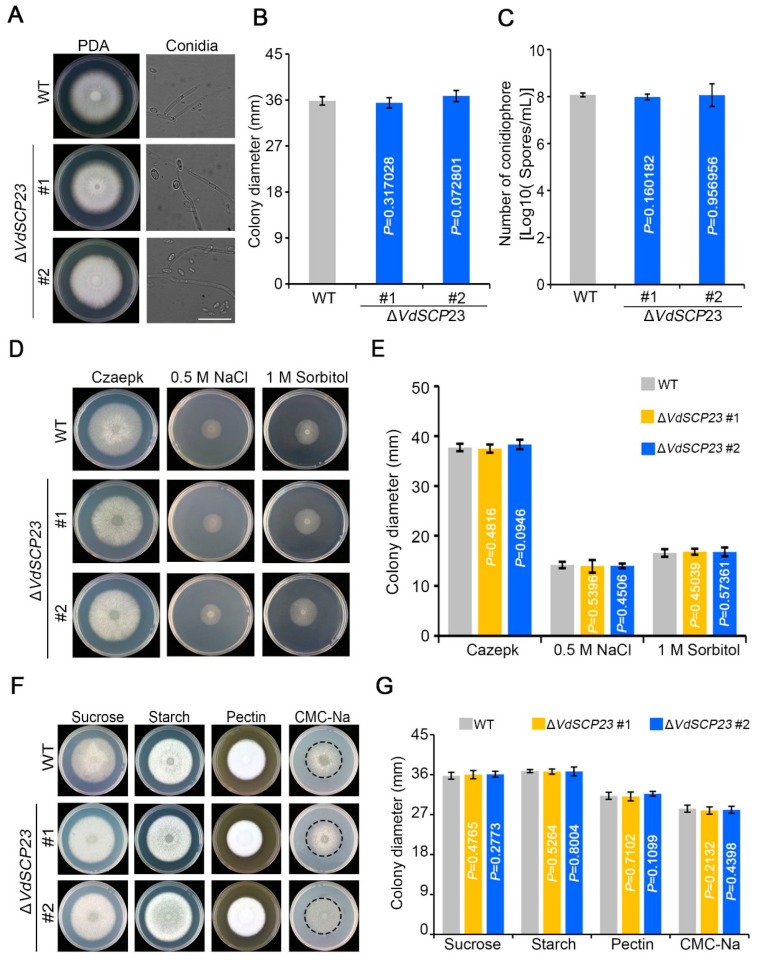
VdSCP23 from *Verticillium dahliae* is not required for growth, normal morphology, stress tolerance or carbon source utilization. (**A**) The growth phenotypes and conidial morphology of Δ*VdSCP23* and wild-type strains grown on PDA plates for 7 days. Scale bar = 20 µm. (**C**) Conidial production of the *VdSCP23* deletion and wild-type strains. (**D**) The growth phenotype of Δ*VdSCP23* and wild-type strains grown on Czapek media containing 0.5 M NaCl or 1 M sorbitol as the osmotic stressor. (**F**) The growth phenotypes of wild-type and Δ*VdSCP23* strains on Capzek media with sucrose, starch, pectin and carboxymethylcellulose sodium (CMC-Na) as carbon sources, respectively. The dashed circle was used to mark the boundaries of colony growth on cellulose containing medium. (**B,G**) Colony diameters of Δ*VdSCP23* and wild-type strains grown on the different media for 7 days. (**E**) Colony diameters of the Δ*VdSCP23* and wild-type strains grown on different media for 9 days. Error bars represent the standard errors. Statistical significance was calculated using Student’s *t* tests.

To establish whether VdSCP23 is involved the morphological development and physiological processes of *V. dahliae*, the phenotypes of the wild-type and Δ*VdSCP23* deletion strains were assayed on PDA plates. Analyses of the *VdSCP23* deletion mutants revealed that there was no obvious difference in colony morphology or growth rate between wild-type and Δ*VdSCP23* strains (Figure 6A,B). The conidial morphology and growth also showed no obvious differences from the wild type or any of the transformants (Figure 6A,C). To investigate whether *VdSCP23* is involved in various stress tolerances, the growth phenotypes of the wild-type and Δ*VdSCP23* strains were examined on Czapek media that imposed osmotic stress (0.5 M NaCl and 1 M sorbitol) and cell-wall-integrity stress (150 μg/mL Congo red and 20 μg/mL calcofluor white (CFW)). The results showed that there was no evident effect on the Δ*VdSCP23* deletion strains compared to the wild-type strain (Figure 6D,E and Figure S6A,B). To detect whether there is any function for *VdSCP23* in the degradation of differing cell wall components, the growth phenotypes of wild-type and Δ*VdSCP23* strains were analyzed on the Capzek media with sucrose, starch, pectin and carboxymethylcellulose (CMC-Na) as carbon sources (Figure 6F,G). These results showed that *VdSCP23* was not required for mycelial growth, morphological development, stress tolerances or carbon source utilization.

### 2.8. VdSCP23 Is Not Required for Virulence

**Figure 7:**
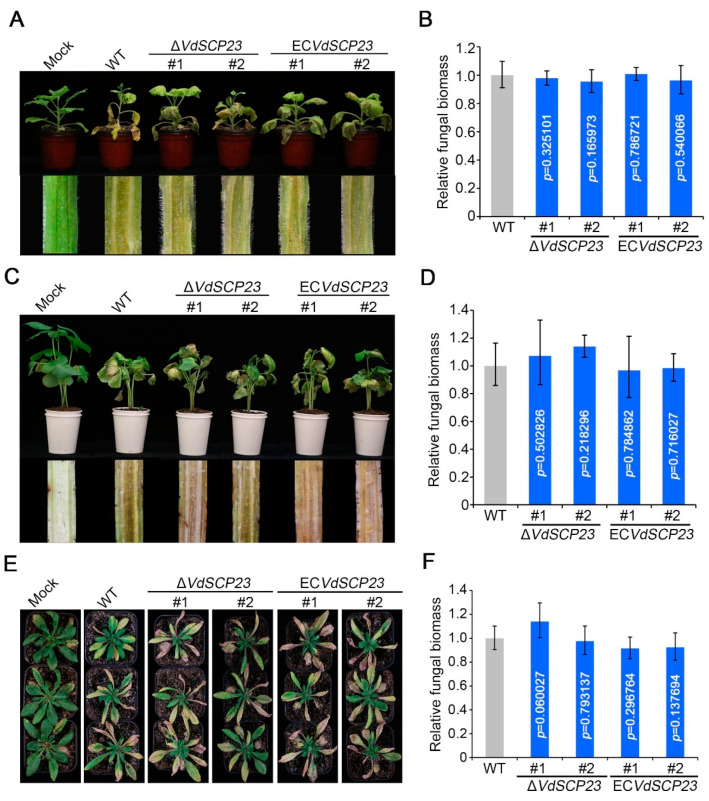
Analyses of the effects of VdSCP23 deletion on virulence of *Verticillium dahliae*. (**A**,**C**,**E**) Four-week-old tobacco was root irrigation-inoculated and three-week-old cotton plants and four-week-old *A. thaliana* seedlings were root dip-inoculated with two independent *VdSCP23* deletion strains and two complemented transformants, while the treatments with the wild-type strain and water served as the positive and negative controls, respectively. The phenotypes were photographed 21 days after inoculation. (**B**,**D**,**F**) Quantification of the fungal biomass of the indicated strains in infected tobacco, cotton and *A. thaliana* by qPCR. Error bars represent the standard error. Statistical significance was calculated using Student’s *t* tests.

To further explore whether *VdSCP23* was associated with *V. dahliae* virulence, the gene expression level of *VdSCP23* during plant infection and the virulence of the deletion mutant Δ*VdSCP23* for plants were analyzed. Expression pattern analysis showed that *VdSCP23* was significantly upregulated during *V. dahliae* infection of cotton roots, especially at two days post-inoculation, with an increase of around 120-fold compared to day 0 (Figure S9). Unexpectedly, virulence assays indicated that the virulence of ΔVdSCP23 was not affected after *VdSCP23* deletion in *N. benthamiana*, cotton or *A. thaliana* seedlings compared to the wild-type strain. Moreover, the complemented strains of *VdSCP23* deletion mutant Δ*VdSCP23* were generated and confirmed by PCR The complemented strains (Figures S7 and S8) showed similar virulence for *N. benthamiana*, cotton and *A. thaliana* seedlings compared to the wild-type and Δ*VdSCP23* mutant strains (Figure 7A,C,E). Furthermore, quantification of the fungal biomass in different plant root and stem tissues confirmed that the colonization of the Δ*VdSCP23* mutant and complemented strains was comparable to the wild type (Figure 7B,D,F). Together, these results showed that, although *VdSCP23* exhibited broad-spectrum immunity suppression, it is not a key virulence factor in *V. dahliae*.

## 3. Discussion

Effectors and SCPs have been identified from a variety of pathogens, and they affect plant immune responses by manipulating various pathways [50]. In this study, through co-expression analysis, we found the small cysteine-rich protein VdSCP23 in *V. dahliae* has broad-spectrum immunity-inhibiting activity. Although VdSCP23 is localized in the plasma membrane, cytoplasm and nucleus, localization in the nucleus does not seem to be essential for its immunity-inhibiting function. VdSCP23 inhibited the immune responses of plants and promoted the infection of *B. cinerea*. Moreover, we determined that the *N-glycosylation* and structural integrity of VdSCP23 are important for its inhibitory function. VdSCP23 did not contribute to the virulence function of *V. dahliae* in several different host plants.

As a secreted protein, VdSCP23 can be secreted extracellularly from *V. dahliae* and enter into host plant cells to perform its function (Figure 2). In *N. benthamiana* cells, VdSCP23 was mainly localized in the cell membrane, cytoplasm and nucleus, even though there was no predicted nuclear localization motif (Figure 2A and Figure 3C). The same localization was observed in natural infection of onion cells and in *A. thaliana* root cells (Figure 3A,B). Adding a nuclear export signal (NES) did not affect the immunosuppressive function of VdSCP23 in *N. benthamiana*, suggesting that the immunosuppressive function of VdSCP23 is independent of its localization to the nucleus but dependent on localization to the cell membrane and cytoplasm. VdSCP23 can inhibit the expression of hypersensitive response, salicylic acid and jasmonic acid signaling genes in plants, thus inhibiting plant immune response. The expression of ethylene, cytokinin and abscisic acid signaling pathway genes will be explored in our future work. Additionally, posttranslational modification determines the activity, stability, localization and interaction of proteins and can also activate or inhibit signal transduction [48], and glycosylation is a common post-translational modification in most proteins. Most glycoproteins obtain glycogroups from the endoplasmic reticulum (ER) and Golgi apparatus, thereby enhancing protein stability, promoting protein localization relative to the membrane or extracellular region and mediating protein interactions [51]. VdSCP23 has two such *N*-glycosylation sites (Figure 2A), and mutation of either of them led to a loss in the immune-suppression function (Figure 5C). Therefore, the *N*-glycosylation of VdSCP23 protein is necessary for its immunosuppressive function. This indicates that the *N*-glycosylation of VdSCP23 may alter its localization in cells or affect the recognition of target proteins. Therefore, we speculate that, when cell death-inducing PAMPs or effectors are secreted extracellularly before recognition by cell membrane receptors, which trigger an immune response, the *N*-glycosylation of VdSCP23 localized around the host cell plasmalemma leads to the binding or inactivation of these PAMPs, effectors or receptors on the cell plasmalemma, blocks downstream signal transduction and, ultimately, inhibits the plant immune response.

One of the most important characteristics of SCPs is that they are rich in cysteine residues, and cysteine residues are the basis for the formation of disulfide bonds [52]. The functions of disulfide bonds have been elucidated in various pathogenic secretory proteins; for example, Avr2, Avr4 and Avr9 from *Cladosporium fulvum* [53,54,55,56]; VdSCP126 from *V. dahliae* [23]; SsSSVP1 from *Sclerotinia sclerotiorum* [57]; and SnTox1 secreted by *Stagonospora nodorum* [58]. The eight cysteine residues from VdSCP23 were predicted to form four disulfide bonds in pairs, but mutation of any single cysteine or the total cysteine content simultaneously did not affect the ability of VdSCP23 to inhibit immunity (Figure 5A,B). Disulfide bonds formed between cysteine residues in secreted proteins are essential for protein stability, protecting these proteins from degradation in a protease-rich apoplast, thus allowing them to play a role in defense induction and pathogenic functions [18]. This suggests that the stability of the VdSCP23 protein was not affected in a way that compromised its immunosuppressive function. On the other hand, truncation of VdSCP23 at the N- or C-termini led to the loss of their immunosuppressive ability (Figure 5C). Thus, this indicates that the normal function of VdSCP23 depends on the full length and structural integrity of the protein but not protein stability.

SCPs from different pathogens have been identified as having the function of inhibiting or stimulating plant immunity, and these functions are typically related to their respective roles in virulence. SCR96, an SCP from *Phytophthora cactorum*, can trigger plant cell death (PCD) in the Solanaceae and is an important virulence factor in *P. cactorum* [59]. *Ustilaginoidea virens* SCRE1 can inhibit tobacco immune-related responses and enhance the sensitivity of rice to pathogens, which is essential for the full virulence of *U. virens* [60]. FoSsP1 can induce cell necrosis and the outbreak of reactive oxygen species so as to negatively regulate the pathogenicity of *Fusarium oxysporum* f. sp. *cubense* [23,61]. VdSCP7 can activate salicylic acid and jasmonic acid signals to stimulate plant immunity and reduce plant susceptibility. Moreover, its knockout can enhance *V. dahliae* infection, which is an important virulence factor for *V. dahliae* [37]. VdSCP41 targets plant transcription factors to suppress host immunity and is critical to the virulence of *V. dahliae* [39]. In this study, although VdSCP23 showed broad-spectrum activity in inhibiting plant immune responses, it did not contribute to the virulence in different host plants (Figure 7). This was in conflict with our initial expectation that the VdSCP23 deletion mutant would exhibit less virulence than the wild-type strain. On the other hand, previous research has also shown that the conidia production ability of the CgCP1-knockout *Colletotricnum gloeosporioides* strain was significantly decreased, and this led to a significant reduction in pathogenicity [62]. In the observation of the growth phenotype, we found that knockout of VdSCP23 did not affect the colony growth or conidia production (Figure 6). However, this cannot be regarded as the reason that VdSCP23 did not contribute to the virulence of *V. dahliae*. In our previous research, it was found that VdSCP27, VdSCP113 and VdSCP126 can stimulate the immune response in *N. benthamiana*, but deletion of any of them individually did not affect the virulence in host plants. However, when VdSCP27 and VdSCP126 were knocked out simultaneously, the virulence of *V. dahliae* was attenuated [23]. Some fungal effectors are generally considered non-essential for virulence due to redundancy related to function or quantity [13]. Therefore, we speculate that VdSCP23 may have functional redundancy in relation to other effectors in *V. dahliae*.

## 4. Materials and Methods

### 4.1. Cultivation of Microbial Strains and Plant Materials

The highly virulent *V. dahliae* wild-type strain Vd991 isolated from *Gossypium hirsutum* was cultured on potato dextrose agar (PDA) or shaken in the liquid Czapek medium at 25 °C. The *V. dahliae* knockout mutants were cultured on PDA with 50 μg/mL hygromycin (Thermo Fisher Scientific, Waltham, MA, USA). The complemented transformants of *V. dahliae* were cultured on PDA with 50 μg/mL geneticin (Thermo Fisher Scientific). *Botrytis cinerea* strain B05.10 was cultured on PDA at 25 °C. *Agrobacterium tumefaciens* GV3101 (WEIDI, Shanghai, China) and AGL-1 (WEIDI, Shanghai, China) were cultured in Luria-Bertani medium at 25 °C for transient expression experiments and fungal transformations, respectively. All plant materials were grown under a 14 h light/10 h dark photoperiod regime in a greenhouse. Cotton (*Gossypium hirsutum* Junmian No. 1) and *N. benthamiana* were grown for three and four weeks, respectively, at 25 °C. The *Arabidopsis thaliana* ecotype Columbia (Col) was grown for four weeks at 23 °C.

### 4.2. Identification and Phylogenetic Analysis of VdSCP23

The candidate small cysteine-rich protein VdSCP23 was identified and cloned from *V. dahliae* Vd991. The subcellular localization of VdSCP23 was determined bioinformatically using WoLF PSORT [63]. SignalP v. 4.1 (D-score cut-off set to 0.500) [64] was used to identify signal peptides and cleavage sites, while Phobius [65] and TMHMM 2.0 [66] were used for the prediction of transmembrane domains. The InterPro database [67] was applied for confirmation of conserved motifs. Identification of disulfide bonds in VdSCP23 was performed using DiANNA http://clavius.bc.edu/~clotelab/DiANNA/ (accessed on 5 November 2021) [47]. NetNGlyc-1.0 was used to predict the *N*-glycosylation sites [49]. VdSCP23 genotyping was analyzed using the Verticilli-Omics https://db.cngb.org/Verticilli-Omics/ (accessed on 5 November 2021) database. The search for homologous proteins of VdSCP23 was performed using BLASTP with the JGI database https://mycocosm.jgi.doe.gov/mycocosm/home (accessed on 5 November 2021), and the phylogenetic tree was constructed using the neighbor-joining method in MEGA11 software.

### 4.3. Transient Expression in Nicotiana benthamiana

*VdSCP23* and its mutant alleles were cloned from *V. dahliae* Vd991 cDNA using the primers listed in Table S3. To determine the roles of specific residues and domains of VdSCP23 in *V. dahliae*–host interactions, the following mutants were generated: *VdSCP23* without the signal peptide (*VdSCP23*^ΔSP^) and *VdSCP23* with the truncated peptide sequences *VdSCP23*^(1–210)^ and *VdSCP23*^(211–423)^. Similarly, *VdSCP23* mutants with a *C*-terminal fusion nuclear export signal (*VdSCP23-NES*) and a nuclear localization signal (*VdSCP23-NLS*) were also constructed. Site-directed mutagenesis of amino acid residues critical to the function of *VdSCP23* (*VdSCP23^N71G^*, *VdSCP23^N74G^, VdSCP23^CnA^*) was performed using a Fast Mutagenesis System Kit (TransGen, Beijing, China). The sequences were cloned into a PVX vector or PGR107 vector, respectively, using a ClonExpress II One-Step Cloning Kit (Vazyme, Nanjing, China) and transferred to *A. tumefaciens* GV3101 for overnight culture in 28 °C LB medium. The cells were collected using high-speed centrifugation, following which the supernatant was decanted. The cells were resuspended in a salt solution containing 10 mM MgCl2, 10 mM MES and 200 μM acetyl syringone (pH 5.6). After two washes in the salt solution, the OD600 of each cell suspension was adjusted to the desired value. To investigate the inhibitory effect of VdSCP23 on *N. benthamiana* cell death-inducing protein, two *A. tumefaciens* cell suspensions carrying appropriate sequence vectors (the VdSCP23 gene and cell death-inducing gene) were mixed in a ratio of 1:1 (OD600 = 0.7 per cell) and expressed simultaneously and transiently in 4-week-old *N. benthamiana* leaves. The Bcl-2-associated X protein (BAX) and green fluorescent protein (GFP) were used as positive and negative controls, respectively. The symptoms affecting the *N. benthamiana* leaves were monitored from 3 to 7 days during the experiment. To verify the transient expression levels of proteins in *N. benthamiana*, total proteins were extracted using the Plant RIPA lysis buffer (Beyotime) and phenylmethanesulfonyl fluoride (Beyotime, Shanghai, China) from the agro-infiltrated *N. benthamiana* leaves 48 h after inoculation, following the manufacturer’s instructions. The proteins were separated using 12% sodium dodecyl sulfate polyacrylamide electrophoresis gels, and transient protein expression in *N. benthamiana* was verified using anti-FLAG antibody (TransGen Biotech, Beijing, China).

### 4.4. Yeast Signal Sequence Trap System

The function of the predicted signal peptide of VdSCP23 was verified as described previously [43]. The coding sequence of the predicted signal peptide of VdSCP23 was fused in-frame in the pSUC2 vector. The recombinant construct pSUC2::SP^VdSCP23^ was transformed into the yeast strain YTK12 and screened on CMD-W (lacking tryptophan) medium. Positive clones were confirmed by PCR using vector-specific primers (Table S3). The positive transformants were incubated on YPRAA medium containing 2% raffinose. Positive clones were transferred to YPRAA medium for invertase secretion detection, and invertase activities of all yeast strains was determined by testing TTC reduction to the insoluble red product triphenyl formamide. The recombinant YTK12 strain carrying the signal peptide sequence of *Avr1b* (pSUC2::SP^Avr1b^) was used as a positive control, while the untransformed YTK12 strain and the YTK12 strain with an empty pSUC2 vector were used as negative controls.

### 4.5. In Vitro Secretion Activity Assay

To detect the secretory activity of VdSCP23 in vitro, a fused fragment including the *TrpC*-promoter region, the coding sequence of *VdSCP23-GFP* and the *Nos*-terminator was introduced into the vector pCOM [68]. The primer pairs for its amplification are listed in Table S3. The conidia of complemented strain EC*VdSCP23-GFP* were collected from a shake culture maintained in complete medium (CM: 0.6% yeast extract, 0.6% casein acids hydrolysate, 1% sucrose) for 3 days. The conidia were transferred to YEPD liquid medium (1% yeast extract, 2% peptone, 2% glucose) containing cotton root and shaken at 25 °C for 24 h. Acetone at a final concentration of 20% (*w*/*v*) was used to extract total protein from the culture supernatant after storage at −80 °C overnight. The solution was centrifuged at 13,000× *g* for 10 min at 4 °C. The total protein was lysed by adding plant RIPA lysis buffer containing phenylmethanesulfonyl fluoride to the dried precipitate. The target proteins were detected with Western blotting using anti-GFP antibodies (TransGen Biotech, Beijing, China), and anti-actin (TransGen Biotech, Beijing, China), anti-mouse (TransGen Biotech, Beijing, China) and anti-rabbit (TransGen Biotech, Beijing, China) antibodies were used as secondary detection antibodies.

### 4.6. Subcellular Localization Assays

To study the subcellular localization of VdSCP23 in planta, the wild-type gene *VdSCP23*, the gene lacking the region encoding the signal peptide sequence (*VdSCP23*^ΔSP^) and genes with the *C*-terminus fused with the nuclear export signal (*VdSCP23-NES*) or the nuclear localization sequence (*VdSCP23-NLS*) were each introduced into the vector pBin::GFP. *A. tumefaciens* GV3101 strains carrying pBin::*VdSCP23*-GFP, pBin-*VdSCP23*^ΔSP^-GFP, pBin::*VdSCP23-NES*-GFP and pBin::*VdSCP23-NLS*-GFP were infiltrated into 3-week-old *N. benthamiana* leaves. The *A. tumefaciens* GV3101 strain carrying pBin::GFP was used as a control. To further examine the subcellular localization of VdSCP23, onion epidermal cells and *Arabidopsis thaliana* root cells infected with *V. dahliae* expressing with *VdSCP23-GFP* were examined with confocal microscopy (Leica, model TCS SP8). In order to better observe fluorescence, *N. benthamiana* leaves were collected 48 h after transient expression, and onion epidermal cells and *Arabidopsis thaliana* root cells were observed at 5 days after infection with *V. dahliae*. These samples were directly imaged with excitation and emission wavelengths of 580 nm and 510 nm, respectively, for GFP. The excitation wavelength of 340 nm and emission wavelength of 488 nm were used for 4′,6-diamidino-2-phenylindole (DAPI) dye. Plasmolysis was performed by treating leaves with 30% sucrose for 15 min.

### 4.7. ROS and Electrolyte Leakage Detection

The elicitor activity was detected through single infiltration and co-infiltration of VdSCP23 with VdEG1 in *N. benthamiana* leaves. VdEG1 and GFP were used as positive and negative controls, respectively. At 48 h after infiltration in *N. benthamiana* leaves, reactive oxygen species (ROS) in *N. benthamiana* leaves were detected using 3′3-diaminobenzidine (DAB) solution as described previously [69]. Electrolyte leakage assays were performed as described previously [70], and ion conductivity was measured using a conductivity meter with Probe LE703 (Mettler-Toledo, Shanghai, China).

### 4.8. Analyses of Gene Expression Levels

To determine the expression of VdSCP23 during infection, three-week-old cotton seedlings were root dip-inoculated with 1 × 10^7^ conidia/mL *V. dahliae*, and the roots were harvested at different time points. The EASYspin Plus RNA Rapid Extraction Kit (Aidlab Biotech, Beijing, China) including genomic DNA (gDNA) removal procedures was used to isolate total RNA. The cDNA was synthesized using a cDNA synthesis supermix (TransGen Biotech, Beijing, China) following the manufacturer’s instructions. QuanStudio 5 (Thermo Fisher Scientific) was used for detection, and cycling parameters for SYBR (TransGen Biotech, Beijing, China) green-based reverse transcription quantitative PCR (RT-qPCR) included the following: an initial 95 °C denaturation step for 3 min followed by denaturation for 15 s at 95 °C, annealing for 30 s at 60 °C and extension for 20 s at 72 °C for 40 cycles. The *V. dahliae* elongation factor 1-α (*EF-1α*) (Table S3) was used as an endogenous reference. The transient expression samples were also collected 2 days after single or co-infiltration of GFP, VdEG1 and VdSCP23 in *N. benthamiana* leaves for the detection of resistance-related gene expression. The total RNA extraction from the samples and cDNA synthesis were performed as above. The *N. benthamiana* elongation factor 1-α (*NbEF-1α*) (Table S3) was used as an endogenous reference, and the primer pairs for analysis of the expression of resistance-related genes are listed in Table S3. The RT-qPCR was performed with an initial 95 °C denaturation step for 3 min followed by denaturation for 15 s at 95 °C, annealing for 20 s at 60 °C and extension for 15 s at 72 °C for 40 cycles. All the RT-qPCR experiments were repeated with three biological replicates, and each biological replicate experiment contained three technical replicates. Relative transcript levels of different genes among the various samples were evaluated using the 2^−∆∆CT^ method [70].

### 4.9. Fungal Transformations for Gene Deletion and Complementation

Targeted gene deletion constructs were prepared as follows: approximately 1 kb 5′ and 3′ flanking regions of the targeted gene *VdSCP23* were amplified from Vd991 gDNA and introduced into the pGKO2-*Hyg* vector, respectively. To generate complemented transformants, the sequences, including the native promoter region, gene sequence (the wild-type *VdSCP23*) and native terminator region, were amplified and introduced into the binary vector pCOM that carries geneticin resistance [68]. The corresponding amplification primer pairs for generating targeted gene deletion and complementation constructs are listed in Table S3.

The positive recombinant vectors were transferred into *A. tumefaciens* strain AGL-1 for the fungal transformation. Gene deletion and complementation transformants were generated with the *A. tumefaciens*-mediated transformation (ATMT) method described previously [71]. The positive transformants were screened and isolated on PDA plates containing 50 µg/mL hygromycin B for gene deletion or 50 µg/mL geneticin for complementation and verified by PCR with the appropriate test primer pairs (Table S3).

### 4.10. Analyses of Growth and Morphology of the VdSCP23 Deletion Mutants

To examine the effect of *VdSCP23* deletion on the growth and morphology of *V. dahliae*, 5 × 10^6^ conidia/mL suspensions of wild-type Vd991 and Δ*VdSCP23* strains were prepared in advance. The suspension of conidia was inoculated at the center of the PDA medium. Colony and mycelial morphology and conidia morphology and production were observed after seven days of culture at 25 °C. Using Czapek as the base medium, the carbon source was replaced to produce media with different carbon sources. Conidial suspensions were inoculated on these media and cultured at 25 °C for seven days to observe the carbon source utilization of the different *V. dahliae* strains. Osmotic stress responses were observed after conidial suspensions were inoculated on the center of a Czapek medium plate containing 0.5 M NaCl and 1.0 M sorbitol and cultured for nine days at 25 °C. The effect of *VdSCP23* deletion on cell wall integrity was observed after seven days of culture at 25 °C on Czapek medium containing 150 μg/mL Congo red (CR) and 20 μg/mL calcofluor white (CFW).

### 4.11. Virulence Assays

Four-week-old *N. benthamiana* leaves were infiltrated on either side with *A. tumefaciens* GV3101 carrying *VdSCP23* or *GFP*. The centers of the infiltration areas of the leaves were inoculated with a 1 × 1 cm *Botrytis cinerea* disc 12 h after agro-infiltration. The plants were moisturized and placed in a 25 °C incubator for 4 days. The diameter of the lesions was measured.

SYBR green-based qPCR was used to detect the biomass of *B. cinerea* in *N. benthamiana*, with an initial 95 °C denaturation step for 3 min followed by denaturation for 15 s at 95 °C, annealing for 30 s at 60 °C and extension for 20 s at 72 °C for 40 cycles. The *B. cinerea* actin gene was used to quantify fungal colonization and the *N. benthamiana EF-1α* gene served as an endogenous control. The experiment was conducted three times, and the primers are listed in Table S3.

Virulence assays with the *V. dahliae* strains were performed with cotton seedlings, as previously described [71]. The 3-week-old cotton seedlings were inoculated with a 5 × 10^6^ conidia/mL suspension of *V. dahliae* using a root-dipping method [71]. Vascular discoloration of infected cotton was observed in longitudinal sections of the shoots 3 weeks after inoculation. Fungal biomass in cotton was determined with SYBR green-based qPCR as described previously [72] with the primer pairs listed in Table S3. The *V. dahliae EF-1α* gene was used to quantify fungal colonization, and the cotton *18S* gene served as an endogenous control. At least 20 cotton seedlings were inoculated for each treatment, and the experiment was conducted three times. Four-week-old *A. thaliana* plants were inoculated with the same inoculation method as for cotton using a 5 × 10^6^ conidia/mL suspension of *V. dahliae*. The roots of four-week-old *A. thaliana* seedlings were immersed in the spore suspensions for 1 min and then transplanted into flowerpots, and the disease-causing phenotypes were observed 3 weeks later. Fungal biomass in *A. thaliana* was determined with SYBR green-based qPCR as described previously [72] with the primer pairs listed in Table S3. The *V. dahliae EF-1α* gene was used to quantify fungal colonization, and the *A. thaliana UBQ1* gene served as an endogenous control. At least three *A. thaliana* seedlings were inoculated for each treatment, and the experiment was conducted three times. The 3-week-old *N. benthamiana* seedlings were inoculated similarly by root-dipping in a 5 × 10^6^ conidia/mL suspension of *V. dahlia*, and pathogenic phenotypes were observed two weeks after inoculation. Fungal biomass in *N. benthamiana* was determined with SYBR Green-based qPCR as described previously [72] with the primer pairs listed in Table S3. The *V. dahliae EF-1α* gene was used to quantify fungal colonization, and the *N. benthamiana NBEF* gene served as an endogenous control. At least five *N. benthamiana* seedlings were inoculated for each treatment, and the experiment was conducted three times.

### 4.12. Statistical Analysis

The standard errors in all figures were calculated for each treatment with at least three replicates. An unpaired Student’s *t* test was performed to determine statistical significance.

## Data Availability

All the data that support the findings of this study are available in the paper and its Supplementary Materials published online.

## Supplementary Materials

The supporting information can be downloaded at: https://www.mdpi.com/article/10.3390/ijms24119403/s1.

## References

[1] Cook D.E., Mesarich C.H., Thomma B.P. (2015). Understanding plant immunity as a surveillance system to detect invasion. Annu. Rev. Phytopathol..

[2] Altenbach D., Robatzek S. (2007). Pattern recognition receptors: From the cell surface to intracellular dynamics. Mol. Plant-Microbe Interact. MPMI.

[3] Boller T., Felix G. (2009). A renaissance of elicitors: Perception of microbe-associated molecular patterns and danger signals by pattern-recognition receptors. Annu. Rev. Plant Biol..

[4] Meng X., Zhang S. (2013). MAPK cascades in plant disease resistance signaling. Annu. Rev. Phytopathol..

[5] Toruño T.Y., Stergiopoulos I., Coaker G. (2016). Plant-Pathogen Effectors: Cellular Probes Interfering with Plant Defenses in Spatial and Temporal Manners. Annu. Rev. Phytopathol..

[6] Dodds P.N., Rathjen J.P. (2010). Plant immunity: Towards an integrated view of plant-pathogen interactions. Nat. Rev. Genet..

[7] Giraldo M.C., Valent B. (2013). Filamentous plant pathogen effectors in action. Nat. Rev. Microbiol..

[8] Jones J.D., Dangl J.L. (2006). The plant immune system. Nature.

[9] Zipfel C. (2008). Pattern-recognition receptors in plant innate immunity. Curr. Opin. Immunol..

[10] Yuan M., Jiang Z., Bi G., Nomura K., Liu M., Wang Y., Cai B., Zhou J.M., He S.Y., Xin X.F. (2021). Pattern-recognition receptors are required for NLR-mediated plant immunity. Nature.

[11] van Kan J.A., van den Ackerveken G.F., de Wit P.J. (1991). Cloning and characterization of cDNA of avirulence gene avr9 of the fungal pathogen *Cladosporium fulvum*, causal agent of tomato leaf mold. Mol. Plant-Microbe Interact. MPMI.

[12] An Y., Wang J., Li C., Revote J., Zhang Y., Naderer T., Hayashida M., Akutsu T., Webb G.I., Lithgow T. (2017). SecretEPDB: A comprehensive web-based resource for secreted effector proteins of the bacterial types III, IV and VI secretion systems. Sci. Rep..

[13] Sharpee W.C., Dean R.A. (2016). Form and function of fungal and oomycete effectors. Fungal Biol. Rev..

[14] Sonah H., Deshmukh R.K., Bélanger R.R. (2016). Computational prediction of effector proteins in fungi: Opportunities and challenges. Front. Plant Sci..

[15] Stergiopoulos I., de Wit P.J. (2009). Fungal effector proteins. Annu. Rev. Phytopathol..

[16] Chen J.Y., Liu C., Gui Y.J., Si K.W., Zhang D.D., Wang J., Short D.P.G., Huang J.Q., Li N.Y., Liang Y. (2018). Comparative genomics reveals cotton-specific virulence factors in flexible genomic regions in *Verticillium dahliae* and evidence of horizontal gene transfer from *Fusarium*. New Phytol..

[17] Overgaard M.T., Sorensen E.S., Stachowiak D., Boldt H.B., Kristensen L., Sottrup-Jensen L., Oxvig C. (2003). Complex of pregnancy-associated plasma protein-A and the proform of eosinophil major basic protein: Disulfide structure and carbohydrate attachment sites. J. Biol. Chem..

[18] Kamoun S. (2006). A catalogue of the effector secretome of plant pathogenic oomycetes. Annu. Rev. Phytopathol..

[19] Chen X.L., Shi T., Yang J., Shi W., Gao X., Chen D., Xu X., Xu J.R., Talbot N.J., Peng Y.L. (2014). *N*-glycosylation of effector proteins by an α-1,3-mannosyltransferase is required for the rice blast fungus to evade host innate immunity. Plant Cell.

[20] Fang A., Han Y., Zhang N., Zhang M., Liu L., Li S., Lu F., Sun W. (2016). Identification and characterization of plant cell death-inducing secreted proteins from *Ustilaginoidea virens*. Mol. Plant-Microbe Interact. MPMI.

[21] Dong Y., Li Y., Zhao M., Jing M., Liu X., Liu M., Guo X., Zhang X., Chen Y., Liu Y. (2015). Global genome and transcriptome analyses of *Magnaporthe oryzae* epidemic isolate 98-06 uncover novel effectors and pathogenicity-related genes, revealing gene gain and lose dynamics in genome evolution. PLoS Pathog..

[22] Mueller O., Kahmann R., Aguilar G., Trejo-Aguilar B., Wu A., de Vries R.P. (2008). The secretome of the maize pathogen *Ustilago maydis*. Fungal Genet. Biol. FG B.

[23] Wang D., Tian L., Zhang D.D., Song J., Song S.S., Yin C.M., Zhou L., Liu Y., Wang B.L., Kong Z.Q. (2020). Functional analyses of small secreted cysteine-rich proteins identified candidate effectors in *Verticillium dahliae*. Mol. Plant Pathol..

[24] Seitner D., Uhse S., Gallei M., Djamei A. (2018). The core effector Cce1 is required for early infection of maize by *Ustilago maydis*. Mol. Plant Pathol..

[25] Qi M., Link T.I., Müller M., Hirschburger D., Pudake R.N., Pedley K.F., Braun E., Voegele R.T., Baum T.J., Whitham S.A. (2016). A small cysteine-rich protein from the Asian soybean rust fungus, *Phakopsora pachyrhizi*, suppresses plant immunity. PLoS Pathog..

[26] Inderbitzin P., Subbarao K.V. (2014). *Verticillium* systematics and evolution: How confusion impedes *Verticillium* wilt management and how to resolve it. Phytopathology.

[27] Klosterman S.J., Atallah Z.K., Vallad G.E., Subbarao K.V. (2009). Diversity, pathogenicity, and management of *Verticillium* species. Annu. Rev. Phytopathol..

[28] Fradin E.F., Thomma B.P. (2006). Physiology and molecular aspects of *Verticillium* wilt diseases caused by *V. dahliae* and *V. albo-atrum*. Mol. Plant Pathol..

[29] Zhang D.D., Dai X.F., Klosterman S.J., Subbarao K.V., Chen J.Y. (2022). The secretome of *Verticillium dahliae* in collusion with plant defence responses modulates *Verticillium* wilt symptoms. Biol. Rev. Camb. Philos. Soc..

[30] Wang B., Yang X., Zeng H., Liu H., Zhou T., Tan B., Yuan J., Guo L., Qiu D. (2012). The purification and characterization of a novel hypersensitive-like response-inducing elicitor from *Verticillium dahliae* that induces resistance responses in tobacco. Appl. Microbiol. Biotechnol..

[31] Liu T., Song T., Zhang X., Yuan H., Su L., Li W., Xu J., Liu S., Chen L., Chen T. (2014). Unconventionally secreted effectors of two filamentous pathogens target plant salicylate biosynthesis. Nat. Commun..

[32] Gao F., Zhang B.S., Zhao J.H., Huang J.F., Jia P.S., Wang S., Zhang J., Zhou J.M., Guo H.S. (2019). Deacetylation of chitin oligomers increases virulence in soil-borne fungal pathogens. Nat. Plants.

[33] Kombrink A., Rovenich H., Shi-Kunne X., Rojas-Padilla E., van den Berg G.C., Domazakis E., de Jonge R., Valkenburg D.J., Sánchez-Vallet A., Seidl M.F. (2017). *Verticillium dahliae* LysM effectors differentially contribute to virulence on plant hosts. Mol. Plant Pathol..

[34] Gui Y.J., Chen J.Y., Zhang D.D., Li N.Y., Li T.G., Zhang W.Q., Wang X.Y., Short D.P.G., Li L., Guo W. (2017). *Verticillium dahliae* manipulates plant immunity by glycoside hydrolase 12 proteins in conjunction with carbohydrate-binding module 1. Environ. Microbiol..

[35] Gui Y.J., Zhang W.Q., Zhang D.D., Zhou L., Short D.P.G., Wang J., Ma X.F., Li T.G., Kong Z.Q., Wang B.L. (2018). A *Verticillium dahliae* extracellular cutinase modulates plant immune responses. Mol. Plant-Microbe Interact. MPMI.

[36] de Jonge R., van Esse H.P., Maruthachalam K., Bolton M.D., Santhanam P., Saber M.K., Zhang Z., Usami T., Lievens B., Subbarao K.V. (2012). Tomato immune receptor Ve1 recognizes effector of multiple fungal pathogens uncovered by genome and RNA sequencing. Proc. Natl. Acad. Sci. USA.

[37] Zhang L., Ni H., Du X., Wang S., Ma X.W., Nürnberger T., Guo H.S., Hua C. (2017). The *Verticillium*-specific protein VdSCP7 localizes to the plant nucleus and modulates immunity to fungal infections. New Phytol..

[38] Wang D., Zhang D.D., Song J., Li J.J., Wang J., Li R., Klosterman S.J., Kong Z.Q., Lin F.Z., Dai X.F. (2022). *Verticillium dahliae* CFEM proteins manipulate host immunity and differentially contribute to virulence. BMC Biol..

[39] Qin J., Wang K., Sun L., Xing H., Wang S., Li L., Chen S., Guo H.S., Zhang J. (2018). The plant-specific transcription factors CBP60g and SARD1 are targeted by a *Verticillium* secretory protein VdSCP41 to modulate immunity. eLife.

[40] Klosterman S.J., Subbarao K.V., Kang S., Veronese P., Gold S.E., Thomma B.P., Chen Z., Henrissat B., Lee Y.H., Park J. (2011). Comparative genomics yields insights into niche adaptation of plant vascular wilt pathogens. PLoS Pathog..

[41] Zhou B.J., Jia P.S., Gao F., Guo H.S. (2012). Molecular characterization and functional analysis of a necrosis- and ethylene-inducing, protein-encoding gene family from *Verticillium dahliae*. Mol. Plant-Microbe Interact. MPMI.

[42] Lacomme C., Santa Cruz S. (1999). Bax-induced cell death in tobacco is similar to the hypersensitive response. Proc. Natl. Acad. Sci. USA.

[43] Jacobs K.A., Collins-Racie L.A., Colbert M., Duckett M., Golden-Fleet M., Kelleher K., Kriz R., LaVallie E.R., Merberg D., Spaulding V. (1997). A genetic selection for isolating cDNAs encoding secreted proteins. Gene.

[44] Oh S.K., Young C., Lee M., Oliva R., Bozkurt T.O., Cano L.M., Win J., Bos J.I., Liu H.Y., van Damme M. (2009). In planta expression screens of *Phytophthora infestans* RXLR effectors reveal diverse phenotypes, including activation of the *Solanum bulbocastanum* disease resistance protein Rpi-blb2. Plant Cell.

[45] Zipfel C. (2009). Early molecular events in PAMP-triggered immunity. Curr. Opin. Plant Biol..

[46] Kubo M., Imai A., Nishiyama T., Ishikawa M., Sato Y., Kurata T., Hiwatashi Y., Reski R., Hasebe M. (2013). System for stable β-estradiol-inducible gene expression in the moss *Physcomitrella patens*. PLoS ONE.

[47] Ferrè F., Clote P. (2006). DiANNA 1.1: An extension of the DiANNA web server for ternary cysteine classification. Nucleic Acids Res..

[48] Liu C., Talbot N.J., Chen X.L. (2021). Protein glycosylation during infection by plant pathogenic fungi. New Phytol..

[49] Gupta R., Brunak S. (2002). Prediction of glycosylation across the human proteome and the correlation to protein function. Pac. Symp. Biocomput..

[50] Kim K.T., Jeon J., Choi J., Cheong K., Song H., Choi G., Kang S., Lee Y.H. (2016). Kingdom-wide analysis of fungal small secreted proteins (SSPs) reveals their potential role in host association. Front. Plant Sci..

[51] Helenius A., Aebi M. (2004). Roles of N-linked glycans in the endoplasmic reticulum. Annu. Rev. Biochem..

[52] Sevier C.S., Kaiser C.A. (2002). Formation and transfer of disulphide bonds in living cells. Nat. Rev. Mol. Cell Biol..

[53] Luderer R., Takken F.L., de Wit P.J., Joosten M.H. (2002). *Cladosporium fulvum* overcomes *Cf-2*-mediated resistance by producing truncated AVR2 elicitor proteins. Mol. Microbiol..

[54] van den Burg H.A., Westerink N., Francoijs K.J., Roth R., Woestenenk E., Boeren S., de Wit P.J., Joosten M.H., Vervoort J. (2003). Natural disulfide bond-disrupted mutants of AVR4 of the tomato pathogen *Cladosporium fulvum* are sensitive to proteolysis, circumvent *Cf-4*-mediated resistance, but retain their chitin binding ability. J. Biol. Chem..

[55] van den Hooven H.W., van den Burg H.A., Vossen P., Boeren S., de Wit P.J., Vervoort J. (2001). Disulfide bond structure of the AVR9 elicitor of the fungal tomato pathogen *Cladosporium fulvum*: Evidence for a cystine knot. Biochemistry.

[56] Van’t Klooster J.W., Van der Kamp M.W., Vervoort J., Beekwilder J., Boeren S., Joosten M.H., Thomma B.P., De Wit P.J. (2011). Affinity of Avr2 for tomato cysteine protease Rcr3 correlates with the Avr2-triggered Cf-2-mediated hypersensitive response. Mol. Plant Pathol..

[57] Lyu X., Shen C., Fu Y., Xie J., Jiang D., Li G., Cheng J. (2016). A small secreted virulence-related protein is essential for the necrotrophic interactions of *Sclerotinia sclerotiorum* with its host plants. PLoS Pathog..

[58] Liu Z., Zhang Z., Faris J.D., Oliver R.P., Syme R., McDonald M.C., McDonald B.A., Solomon P.S., Lu S., Shelver W.L. (2012). The cysteine rich necrotrophic effector SnTox1 produced by *Stagonospora nodorum* triggers susceptibility of wheat lines harboring *Snn1*. PLoS Pathog..

[59] Chen X.R., Li Y.P., Li Q.Y., Xing Y.P., Liu B.B., Tong Y.H., Xu J.Y. (2016). SCR96, a small cysteine-rich secretory protein of *Phytophthora cactorum*, can trigger cell death in the *Solanaceae* and is important for pathogenicity and oxidative stress tolerance. Mol. Plant Pathol..

[60] Zhang N., Yang J., Fang A., Wang J., Li D., Li Y., Wang S., Cui F., Yu J., Liu Y. (2020). The essential effector SCRE1 in *Ustilaginoidea virens* suppresses rice immunity via a small peptide region. Mol. Plant Pathol..

[61] Wang Y., Zhang X., Wang T., Zhou S., Liang X., Xie C., Kang Z., Chen D., Zheng L. (2022). The small secreted protein FoSsp1 elicits plant defenses and negatively regulates pathogenesis in *Fusarium oxysporum* f. sp. *cubense* (Foc4). Front. Plant Sci..

[62] Wang W., An B., Feng L., He C., Luo H. (2018). A *Colletotrichum gloeosporioides* cerato-platanin protein, CgCP1, contributes to conidiation and plays roles in the interaction with rubber tree. Can. J. Microbiol..

[63] Horton P., Park K.J., Obayashi T., Fujita N., Harada H., Adams-Collier C.J., Nakai K. (2007). WoLF PSORT: Protein localization predictor. Nucleic Acids Res..

[64] Petersen T.N., Brunak S., von Heijne G., Nielsen H. (2011). SignalP 4.0: Discriminating signal peptides from transmembrane regions. Nat. Methods.

[65] Käll L., Krogh A., Sonnhammer E.L. (2007). Advantages of combined transmembrane topology and signal peptide prediction--the Phobius web server. Nucleic Acids Res..

[66] Krogh A., Larsson B., von Heijne G., Sonnhammer E.L. (2001). Predicting transmembrane protein topology with a hidden Markov model: Application to complete genomes. J. Mol. Biol..

[67] Apweiler R., Attwood T.K., Bairoch A., Bateman A., Birney E., Biswas M., Bucher P., Cerutti L., Corpet F., Croning M.D. (2001). The InterPro database, an integrated documentation resource for protein families, domains and functional sites. Nucleic Acids Res..

[68] Zhou L., Zhao J., Guo W., Zhang T. (2013). Functional analysis of autophagy genes via *Agrobacterium*-mediated transformation in the vascular wilt fungus *Verticillium dahliae*. J. Genet. Genom..

[69] Bindschedler L.V., Dewdney J., Blee K.A., Stone J.M., Asai T., Plotnikov J., Denoux C., Hayes T., Gerrish C., Davies D.R. (2006). Peroxidase-dependent apoplastic oxidative burst in *Arabidopsis* required for pathogen resistance. Plant J. Cell Mol. Biol..

[70] Oh C.S., Pedley K.F., Martin G.B. (2010). Tomato 14-3-3 protein 7 positively regulates immunity-associated programmed cell death by enhancing protein abundance and signaling ability of MAPKKK α. Plant Cell.

[71] Liu S.Y., Chen J.Y., Wang J.L., Li L., Xiao H.L., Adam S.M., Dai X.F. (2013). Molecular characterization and functional analysis of a specific secreted protein from highly virulent defoliating *Verticillium dahliae*. Gene.

[72] Santhanam P., van Esse H.P., Albert I., Faino L., Nürnberger T., Thomma B.P. (2013). Evidence for functional diversification within a fungal NEP1-like protein family. Mol. Plant-Microbe Interact. MPMI.

