# Supplementary material for: The Verticillium dahliae Small Cysteine-Rich Protein VdSCP23 Manipulates Host Immunity
Source: Int J Mol Sci. 2023 May 28;24(11):9403. doi: 10.3390/ijms24119403 (PMC10253731; doi:10.3390/ijms24119403)
Supplement: Supplementary file 1 [file ijms-24-09403-s001.zip › Supplemental_Figures.pdf]

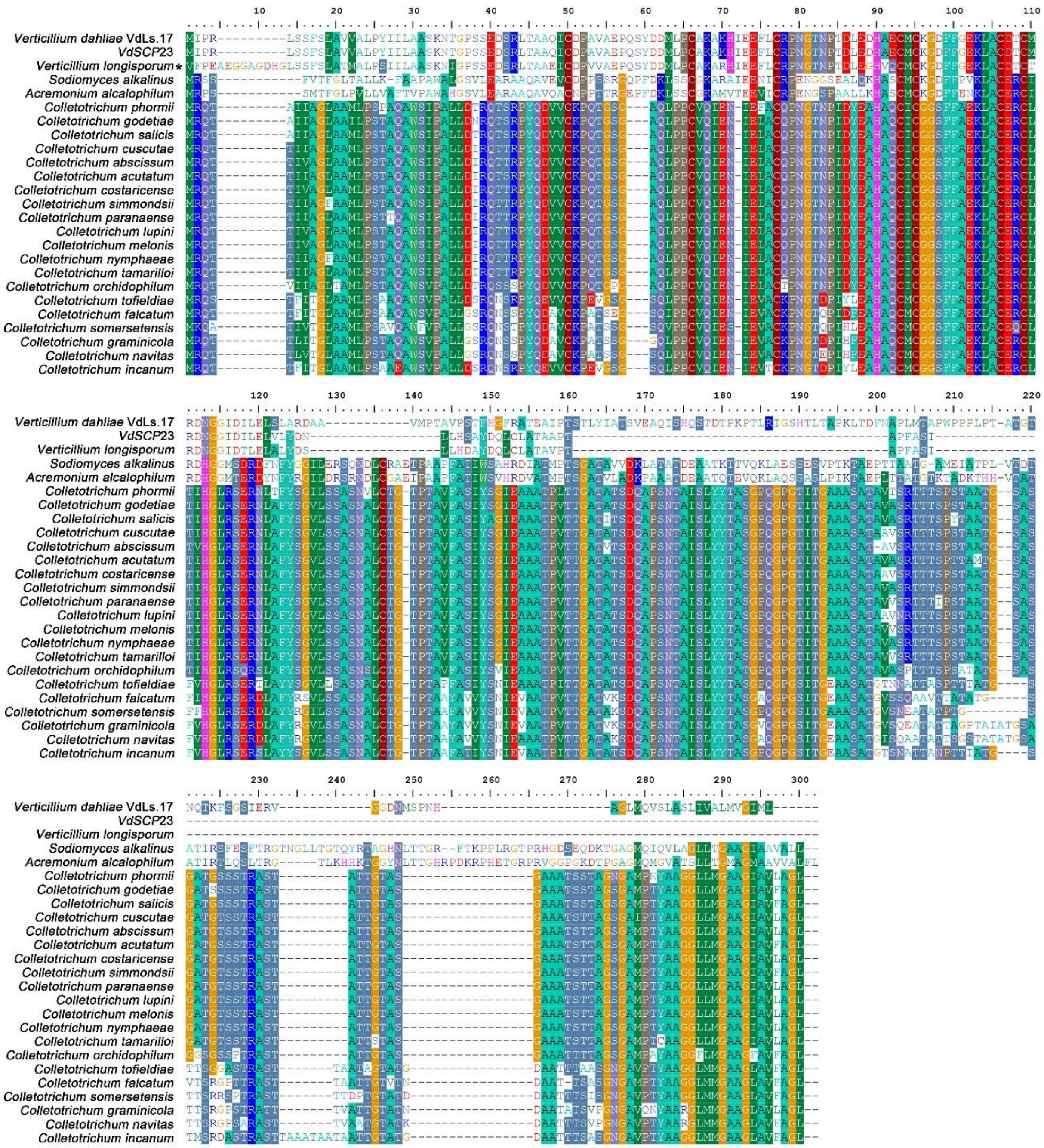

Figure S1 | Alignment of all homologous VdSCP23 sequences for phylogenetic analysis. Asterisk represents the first 414 unmatched amino acids in the sequence.

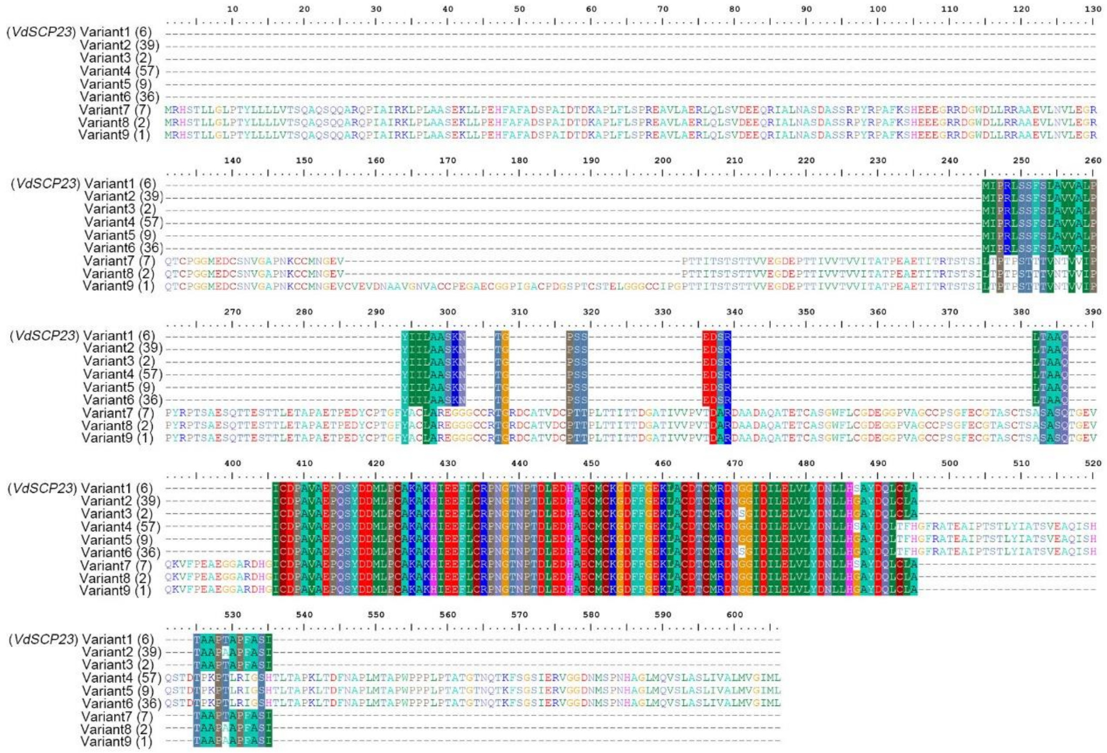

Figure S2 | Genotypic analysis of VdSCP23 in 159 strains *Verticillium dahliae* from Verticilli-Omics database (https://db.cngb.org/Verticilli-Omics/). VdSCP23 belongs to variant 1, numbers within parentheses represent the number of strains in each variant group.

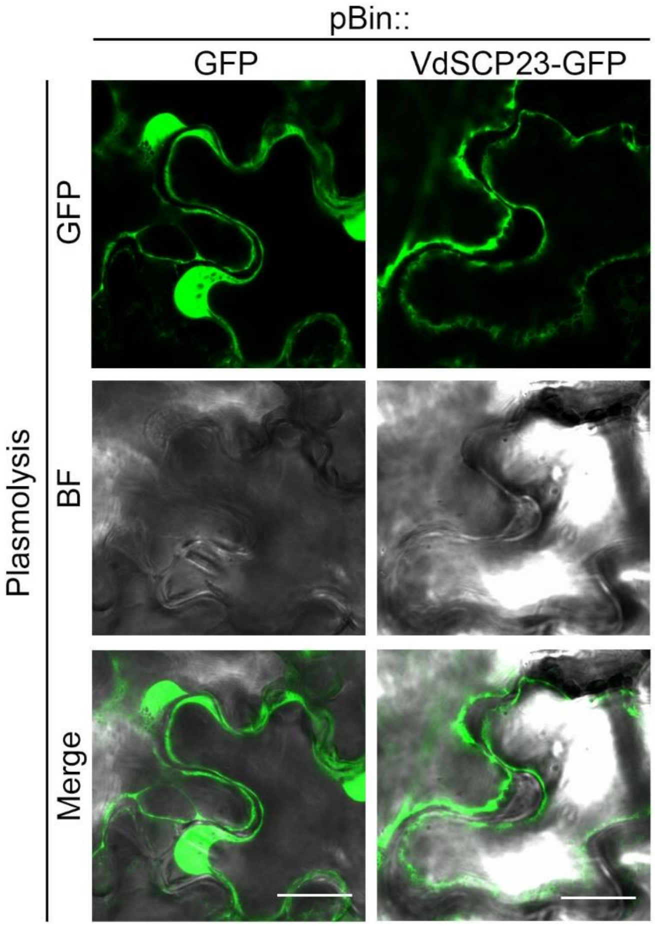

Figure S3 | Localization of VdSCP23 in *N. benthamiana* leaves after plasmolysis. Localization of VdSCP23 in tobacco leaf cells treated with 30% sucrose, the localization of GFP expression was used as control. Bars = 2 μm.

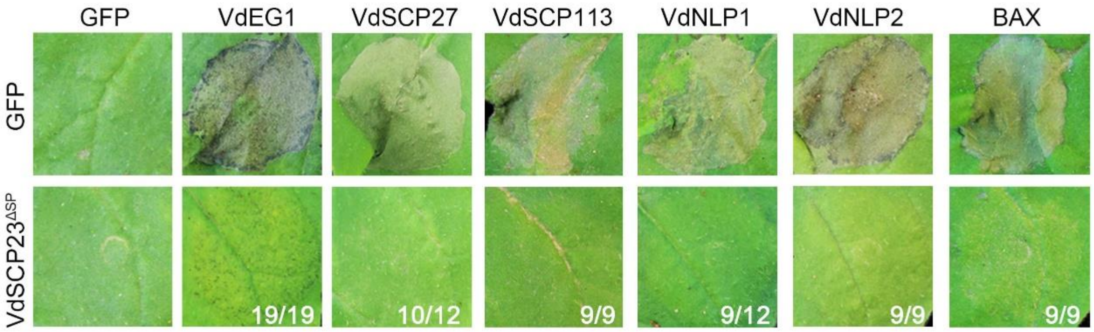

Figure S4 | Analysis of the inhibitory activity of VdSCP23 lacking the signal peptide against the cell death-induced proteins. VdSCP23$^{\Delta SP}$ was co-agroinfiltrated with eight different *Verticillium dahliae* cell death-induced proteins. Co-expression of VdSCP23$^{\Delta SP}$ with GFP or BAX served as controls. Phenotypes were observed 6 days post-agro-infiltration. The numerator at the bottom of leaves represents the number suppressing cell death out of the total number of tested leaves (denominator).

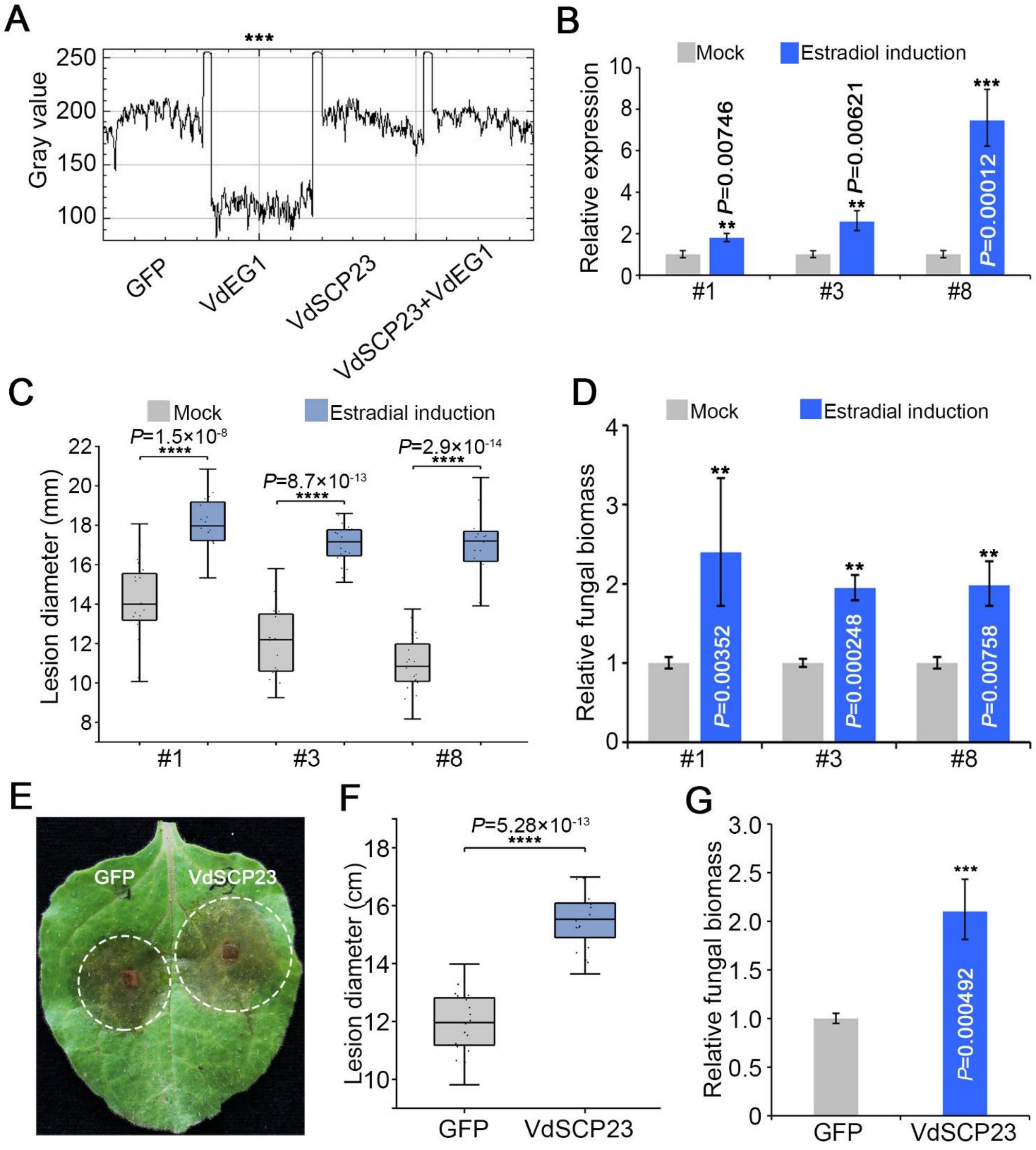

Figure S5 | *Verticillium dahliae* protein VdSCP23 enhances host susceptibility by suppressing immunity. (A) Quantitative analysis of gray value of tobacco leaves under different treatments. **(B)** Expression of *VdSCP23* gene in transgenic *N. benthamiana* induced by estradiol. #1, #3 and #8 represent the three transgenic lines. **(C) and (D)** The lesion diameters and biomass of *Botrytis cinerea* infection of *N. benthamiana* leaves including estradiol-induced and non-estradiol-induced transgenic

lines. **(E)** Disease symptoms of *B. cinerea* on *N. benthamiana* leaves of transient expression of VdSCP23. **(F) and (G)** Disease phenotypes of *B. cinerea* on *N. benthamiana* leaves was evaluated at 4 days post-inoculation by determining the lesion diameters, and fungal biomass was determined by quantitative PCR (qPCR). Error bars represent standard errors of the mean. **, *** and **** indicate statistical significance at $P < 0.01$, $P < 0.001$ and $P < 0.0001$ according to the Student's *t* tests.

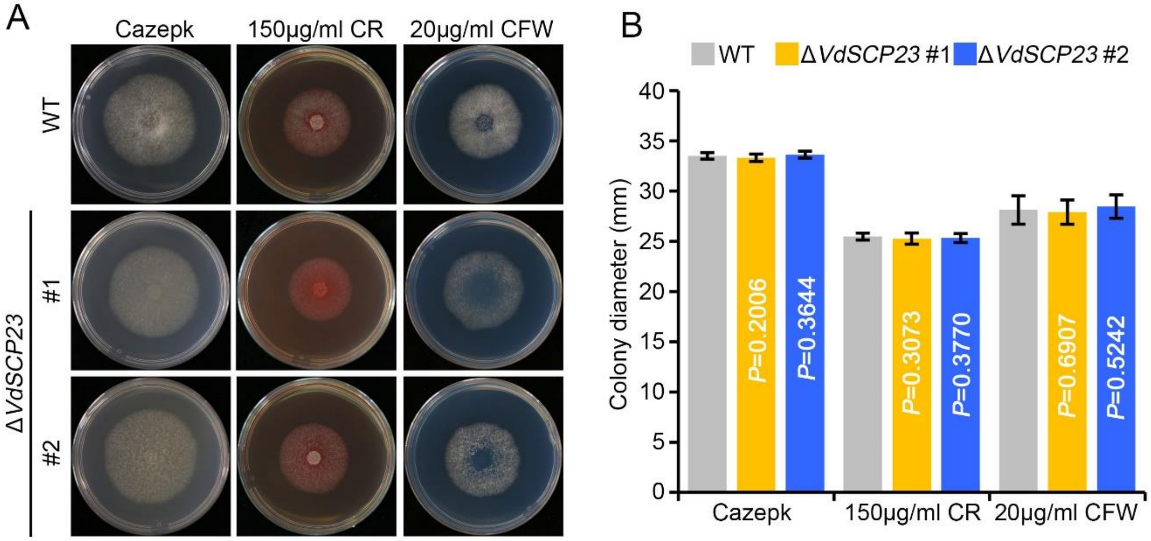

Figure S6 | *VdSCP23* deletion does not affect cell wall stress. (A) The growth of *VdSCP23* deletion and wild-type strains on Czapek-Dox plates containing CR as 150 µg/mL or CFW as 20 µg/mL. **(B)** The colony diameters of different strains were determined from five plates in each experiment and the experiment was repeated three times. Statistical significance was calculated using the Student's *t* tests.

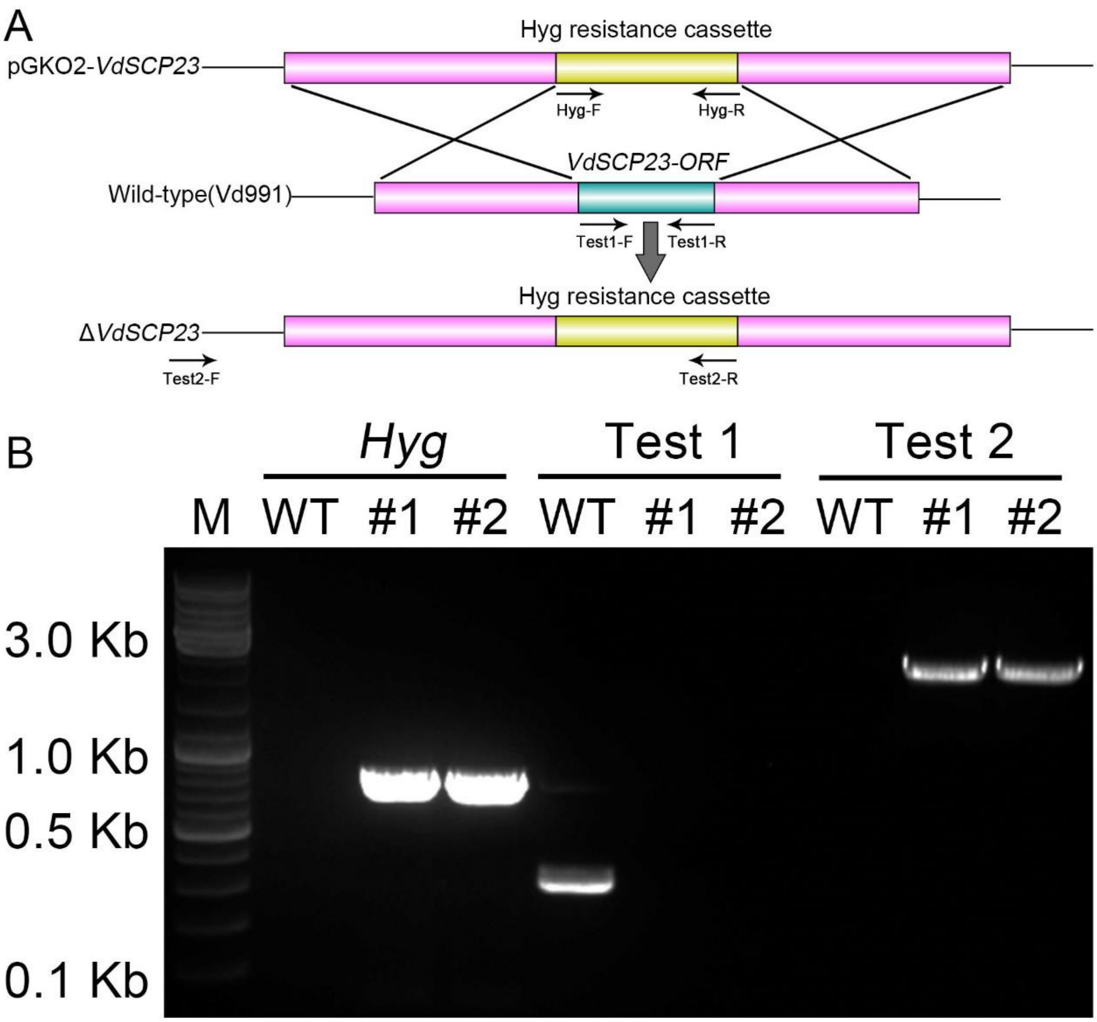

Figure S7 | The verification for *VdSCP23* deletion mutants by polymerase chain reaction (PCR). (**A**) Outline of the procedure followed for gene knockout via homologous recombinant. (**B**) PCR analysis for gene deletion transformants. Hyg: PCR amplification of the positive selection marker hygromycin phosphotransferase gene. Test 1: the internal detection primer designed according to *VdSCP23* gene sequence. Test 2: PCR amplification of combination for the outer sequence upstream of target gene and the inner sequence of hygromycin phosphotransferase gene. WT: the genome of Vd991 of wild type *V. dahliae*. M: DNA ladder used as a size marker.

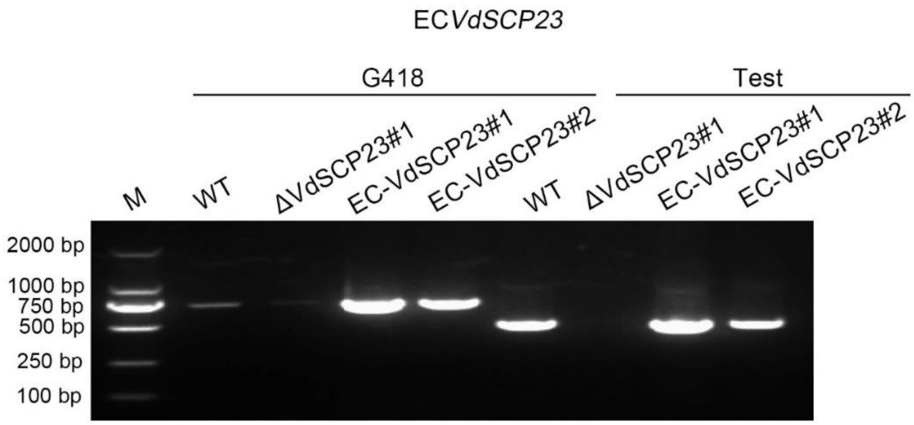

Figure S8 | Polymerase chain reaction (PCR) analysis of complemented transformants. G418: PCR amplification of the positive selection marker geneticin phosphotransferase gene. Test: PCR amplification of the internal gene sequence of *VdSCP23*. WT: wild type Vd991. M: DNA ladder used as a size marker.

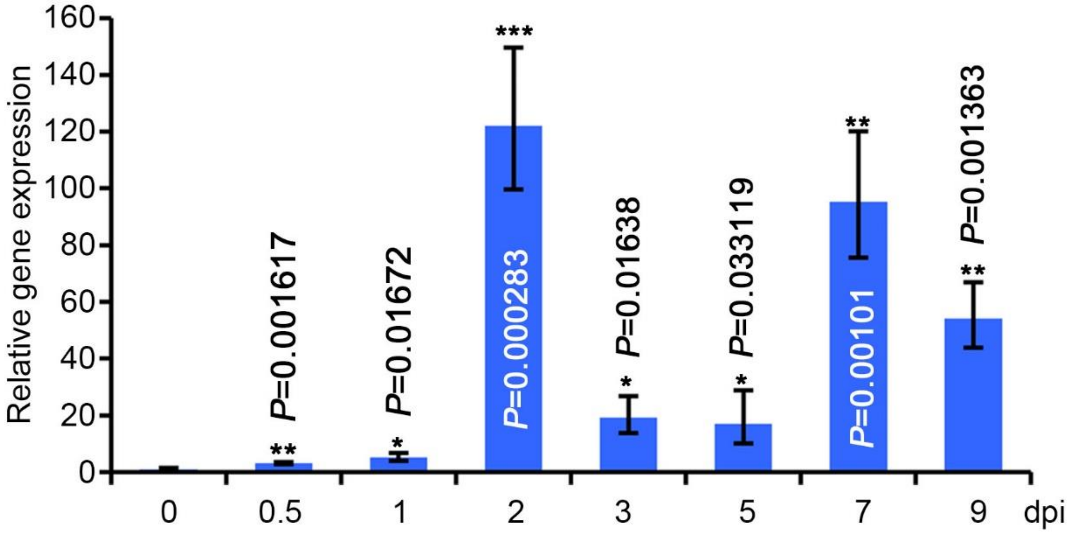

Figure S9 | Expression of *VdSCP23* during infection of cotton roots. Wild-type *V. dahliae* was used to infect 3-week-old cotton plants (Junmian 1), and cotton roots were harvested at different time points after inoculation. Reverse transcription-quantitative PCR was performed to determine the expression levels of *VdSCP23* relative to *V. dahliae EF-1α*. Error bars represent standard errors of the mean. *, ** and *** indicate statistical significance at $P < 0.05$, $P < 0.01$ and $P < 0.001$ according to the Student's *t* tests.
